# Influence of Admission Pathways on Learning Strategies, Assessment Engagement, and Academic Performance Among First-Year Medical Students: Mixed Methods Retrospective Observational and Cross-Sectional Survey Study

**DOI:** 10.2196/68636

**Published:** 2026-02-02

**Authors:** Issarawan Keadkraichaiwat, Chantacha Sitticharoon, Punyapat Maprapho, Nisa Jangboon, Nadda Wannarat

**Affiliations:** 1 Faculty of Medicine Siriraj Hospital, Mahidol University, 2 Wanglang Rd, Siriraj, Bangkoknoi, 10700, Thailand, 66 80-992-4536

**Keywords:** admission systems, self-regulated learning strategies, assessment engagement statistics, academic outcome, course learning outcome, nongrading evaluation

## Abstract

**Background:**

Medical school admission pathways are designed to select suitable applicants, with different approaches potentially impacting students’ learning behaviors and performance.

**Objective:**

This study aimed to compare students’ self-regulated learning (SRL) strategies, assessment engagement statistics (AES), nongrading evaluation (Outstanding [“O”]/Satisfactory [“S”]/Unsatisfactory [“U”]) preferences, and academic performance across admission pathways, and analyze correlations and linear regression models among summative scores, AES, and course learning outcome (CLO) scores.

**Methods:**

This mixed methods retrospective observational and cross-sectional survey study used census sampling with selection criteria of all enrolled first-year medical students in 2021 (N=319) across 4 admission pathways: academic (n=23), quota (n=6), test (n=261), and rural (n=29). Demographics included age (19‐24 years) and sex (167/319, 52.4% male). AES, CLO scores, and summative scores were obtained from institutional databases. Two system-embedded institutional questionnaires assessed SRL strategies (316/319, 99.1% response rate) and “O”/“S”/“U” preferences (299/319, 93.7% response rate). Outcome measures included SRL strategies, AES, “O”/“S”/“U” preferences, CLO scores, and summative scores. Statistical significance was set at *P*<.05.

**Results:**

When compared among pathways, using one-way ANOVA with Fisher least significant difference post hoc tests, the academic group reported significantly higher mean (with 95% CI) goal setting (4.35, 4.07‐4.63), enthusiasm (4.43, 4.18‐4.69), and lower stress during study (2.64, 2.15‐3.12), while the rural group showed higher pre-examination stress (4.38, 4.10‐4.66) (all *P*<.05). Most academic (14/22, 63.6%), quota (5/6, 83.3%), and test students (132/243, 54.3%) preferred “O”/ “S”/“U,” while the rural students preferred “S”/“U” (13/28, 46.4%). The academic group showed significantly higher CLO and summative scores but fewer total and intentional attempts and instances of first-pass and highest scoring attempts (all *P*<.05), whereas the rural group showed significantly lower CLO and summative scores and higher instances of first-pass and highest scoring attempts (all *P*<.05). For correlation analyses, using Pearson correlation coefficient, summative scores were positively correlated with CLO scores and number of passings and negatively with first-pass attempts. For multiple linear regression analyses, summative scores were positively influenced by number of passings for each CLO and CLO scores and negatively influenced by instances of first-pass attempts and highest scoring attempts. Overall, the academic group demonstrated higher academic performance and fewer attempts and instances of first-pass and highest scoring attempts, while the rural group showed lower academic performance, requiring more attempts for first-passing CLOs.

**Conclusions:**

Admission pathways significantly influence students’ SRL strategies, AES, evaluation preferences, and academic performance. This study is innovative in analyzing these interconnected components within a single cohort, unlike prior research that examined them separately. By integrating assessment-engagement analytics with SRL data, it offers equity-oriented evidence on how admission systems shape learning behaviors and academic trajectories. These findings provide actionable insights for inclusive curriculum design and early identification of at-risk students. Real-world implications include targeted mentoring, SRL-focused interventions, and assessment reforms balancing academic rigor with psychological safety.

## Introduction

To address the diversity of medical curricula, institutions use various admission pathways, which may impact students’ academic outcomes, self-regulated learning (SRL) strategies, assessment engagement statistics, and evaluation preferences. In comparisons among admission pathways, there were differences in academic outcomes and the likelihood of on-time graduation [1]. In terms of academic performance, students admitted through the Open National Unified Admission track, characterized by the highest competitiveness and a reserved quota for underprivileged students, predominantly comprising children of military personnel and government employees within the Ministry of Education in Jordan, had significantly higher graduate grade point average (GPA) than those in other tracks, whereas students from the other pathway track and an international track exhibited the lowest graduating GPA [1]. Furthermore, students entering through the GENERAL admission pathway in New Zealand demonstrated significantly higher GPAs in years 2‐3 than students admitted through the Māori and Pacific Admission Scheme, which was designed to address shortages in the Māori and Pacific health workforce, reduce significant disparities in health outcomes, and uphold the rights of Māori within the New Zealand pathway [2]. In addition, multiple regression analysis demonstrated that the undergraduate entry pathway positively contributed to GPA in years 2‐3 in New Zealand [2].

For students from various academic backgrounds, health-related undergraduate degree students consistently surpassed those from BioMed, Science, Humanities, and Business undergraduate degree programs in performance on medical science, clinical practice, objective structured clinical examinations, and overall performance when compared to other pathways in Australia [3].

For graduation rates, students admitted through the competitive Open National Unified Admission track and the reserved quota for underprivileged student track achieved the highest percentage of on-time graduation, followed by the children of university staff track in Jordan [1]. Similarly, students who gained admission through the GENERAL pathway in New Zealand exhibited a greater likelihood of completing their intended program than those from the Māori and Pacific Admission Scheme pathway [2].

Admission to the Faculty of Medicine Siriraj Hospital in the 2021 academic year followed the Thai University Central Admission System (TCAS) framework and aligned with the broader educational policy goals of Thailand’s national medical school admission framework, which emphasizes not only academic merit but also equity, inclusion, and workforce distribution [4].

Importantly, at the time of the 2021 admission cycle, TCAS is structured as 4 sequential rounds and operates as a binding system, that is, once students are accepted and confirm their place in an earlier round, they cannot participate in subsequent rounds. This structure ensures efficient allocation, reduces redundancy, and facilitates early selection of high-priority candidates. Round 1 emphasizes portfolio submission and interviews, making it suitable for applicants with strong academic records and extracurricular achievements, while round 2 follows a quota system designed for students from specific regions or with special talents. Round 3 relies on standardized examinations such as the Thai General Aptitude Test, the Thai Professional Aptitude Test (TPAT), and core subjects, favoring applicants with strong test performance, whereas round 4 enables institutions with unfilled seats to admit students through their own criteria, offering opportunities for applicants not accepted in earlier rounds [5].

At our institution, via 3 TCAS rounds, 4 pathways (groups) were established: round 1—the academic group targeting high-potential students with demonstrated cognitive strengths and scientific aptitude, particularly those participating in national science Olympiads; round 2—the quota group, promoting diversity by admitting students with exceptional talents in music or sports and individuals holding a bachelor’s degree; and round 3—the central admission test pathway (test group) and the pathway for students required to serve as rural doctors (rural group), prioritizing academic achievement through competitive entrance examinations [5,6]. Notably, students in the rural group were officially enrolled under the Faculty of Medicine, Praboromarajchanok Institute, but undertook their preclinical study together with students of the Faculty of Medicine Siriraj Hospital.

Differences in admission pathways may be associated with variations in students’ SRL behaviors. According to Zimmerman’s cyclical model, SRL encompasses 3 interrelated phases: the forethought phase, which involves goal setting, task analysis, and motivational beliefs; the performance phase, which includes self-control, monitoring, and strategy implementation; and the self-reflection phase, which focuses on self-evaluation and adaptive reactions after learning tasks [7]. SRL may influence students across various admission systems, since it influences how they approach learning tasks, manage challenges, and engage with evaluation, and plays a crucial role in their academic success, particularly among students with different backgrounds [8].

In addition, nongrading or mastery-based systems, such as pass/fail or pass/satisfactory/outstanding formats, have gained attention for their potential to reduce stress [9], promote intrinsic motivation [10-12], and foster deeper, self-directed learning [10]. These approaches are especially relevant in high-stakes disciplines such as medicine, where long-term competence and well-being are critical [13], probably reducing competitiveness and anxiety while promoting equity and fostering collaborative learning environments [10,14,15]. Despite the growing adoption of nongrading evaluation systems in medical education as part of curricular reform, limited evidence exists on how students from different admission tracks perceive these systems or how such reforms influence their SRL strategies and academic performance.

Student feedback is widely recognized as a crucial mechanism for curriculum evaluation and improvement, serving purposes such as quality assurance, course and program revision, and the evaluation of teaching quality and assessment tasks, while also being critical to student learning by influencing motivation, engagement, self-reflective learning, and overall performance, as well as supporting iterative refinement and long-term acceptance [16-19]. Understanding the perceptions of students from different admission pathways is particularly important, as their diverse educational, social, and cultural backgrounds may shape how they interpret and adapt to new evaluation systems, potentially influencing not only their motivation and learning behaviors but also the effectiveness of these systems in supporting diverse student cohorts [20].

Comparisons of academic outcomes among various admission pathways have been partly reported; however, comparisons of preclinical students’ SRL strategies, assessment engagement statistics, preferences regarding nongrading evaluation, and course learning outcomes (CLOs) and summative scores have not been studied. This study aimed to (1) compare students’ SRL strategies, assessment engagement statistics, preferences regarding nongrading evaluation, and academic performance among the academic, quota, test, and rural groups; (2) determine correlations among summative scores, students’ assessment engagement statistics, and CLO scores in each group; and (3) identify factors contributing to summative scores in each group through multiple linear regression analyses. By addressing these gaps, our study provides a comprehensive understanding of how admission pathways may shape not only academic outcomes but also learning behaviors, essential for designing inclusive medical curricula that support all learners [7].

## Methods

### Study Design

This study used a mixed methods design consisting of a retrospective observational component and a cross-sectional survey component. The retrospective observational component included assessment engagement statistics and CLO scores obtained from the Siriraj E-Learning and Education Community, as well as summative examination scores retrieved from the Siriraj Campus Management System (SiCMs). The cross-sectional component consisted of 2 system-embedded institutional questionnaires: questionnaire 1 assessing students’ SRL strategies and questionnaire 2 assessing preferences regarding nongrading evaluation, both administered within the SiCMs platform. The integration of quantitative and qualitatively informed data enabled a comprehensive examination of learning behaviors and academic performance across admission pathways.

Although this study adopted a mixed methods framework, the qualitative component was limited to a qualitatively informed instrument development process based on literature review and expert input rather than primary qualitative data collection. Therefore, formal qualitative data saturation was not applicable. Instead, content validity was ensured through iterative expert review and consensus to confirm comprehensive coverage of relevant educational constructs.

### Study Protocol

Participants included all first-year medical students enrolled in the academic year 2021 at the Faculty of Medicine Siriraj Hospital, Mahidol University, using a census sampling approach. Selection criteria included all officially enrolled students, with no exclusions applied.

### Ethical Considerations

This study used routinely collected educational data and system-embedded questionnaires. The research protocol was reviewed and approved by the Siriraj Institutional Review Board. The initial protocol was granted exemption from full review under protocol number 370/2565 (exempt). A subsequent amendment, which included additional demographic data (age, school region, and hometown region), was approved under certificate of approval number 731/2025. For the retrospective observational component, the institutional review board granted a waiver of written informed consent because the data were obtained from existing institutional databases, posed minimal risk, and were analyzed in deidentified, aggregate form. For the cross-sectional component, participation involved completion of system-embedded questionnaires as part of routine institutional processes; therefore, no additional consent was required for secondary analysis of anonymized data, as approved by the institutional review board. All datasets were anonymized prior to export, with direct identifiers removed, and were stored on password-protected computers with restricted access. No financial or academic incentives were provided, and participation had no impact on academic standing. No identifiable images or sensitive personal information was included in the analysis.

### Admission to the Faculty of Medicine Siriraj Hospital

For the academic group, applicants had to be high school students who had achieved gold or silver medals at the National Academic Olympiad in the following fields: Physics, Chemistry, Biology, Mathematics, Computer Science (Informatics), Geography, Astronomy, and Astrophysics; or have completed Camp 2 in Earth and Space Science. In addition, applicants could be high school students in the year of the training camp or those selected to attend the Academic Olympiad Training Camp Round 1 in Biology, Chemistry, Physics, Computer Science, or Mathematics. Furthermore, they were required to have a GPA of at least 3.00 in Science, Mathematics, English, Thai, and Social Studies. Selection was primarily based on portfolio assessment, including activities, academic achievements, and other special skills or talents, as well as a statement of purpose. Subsequently, they underwent multiple mini-interviews (MMIs), which were constructed based on the conceptual framework of the objective structured clinical examination [21], and final selection was determined according to MMIs criteria.

For the quota group, applicants must (1) have talent in sports or music at national or international levels; (2) have eligibility for the Mahidol Medical Scholars Program, with a GPA of at least 3.00 in Science, Mathematics, English, Thai, and Social Studies; or (3) be holding a bachelor’s degree with a GPA of at least 3.25 in any field in Thailand, with age not exceeding 35 years. Applicants were primarily selected based on academic examinations organized by the TCAS, accounting for 70%, including 20% Mathematics, 40% Sciences (Physics, Chemistry, and Biology), 20% English, 10% Thai, and 10% Social Studies, as well as 30% Ordinary National Educational Test for music talent, or General Aptitude Test/Professional Aptitude Test for sport talents and Mahidol Medical Scholar Program, or the TPAT organized by the Consortium of Thai Medical Schools for bachelor’s graduates. Finally, they underwent MMIs for definitive selection.

For the test group, applicants were high school students selected through academic examinations weighted at 70% TCAS and 30% TPAT, as described for the quota group, along with a general interview, including both a standard personal interview and a psychiatric assessment, as well as a health examination, to screen out individuals unsuitable for medical studies. The eligibility criteria for passing these assessments were based on the national medical school admission standards, which ensure that applicants are free from any physical or mental health conditions that could hinder their medical education, clinical training, or future medical practice. These criteria are defined in the official 2016 regulation on student eligibility and include, for example, severe psychiatric disorders, active communicable diseases, significant physical disabilities, and uncorrectable hearing or visual impairments.

For the rural group, high school students were admitted under Thailand’s national “Doctors for Rural Areas” policy, implemented through the Collaborative Project to Increase Production of Rural Doctors, which was established to address physician shortages in underserved areas and to reflect Thailand’s national “Doctors for Rural Areas” policy, aiming to promote equity in health care distribution [22]. Students from designated provinces must have their names in the house records of the specified province continuously for not less than 5 years, attend a school in the designated area, and receive government financial support throughout medical training. Upon graduation, graduates are obligated to return to their home province or designated health region to work in public hospitals as part of a binding service requirement [22]. They are required to serve 3 years in Ministry of Public Health hospitals, with a financial penalty of approximately US $11,300 imposed for noncompliance [22]. They were selected based on a GPA of at least 3.00, a 70% academic examination score through TCAS as the test group, and 30% interview performance. Although they were officially enrolled under the Faculty of Medicine, Praboromarajchanok Institute, they studied their preclinical years alongside the Faculty of Medicine Siriraj Hospital’s students, receiving the same curriculum delivery, teaching and assessment processes, access to facilities and equipment, academic and mentoring support, and extracurricular opportunities. After finishing the preclinical curriculum, they continued their clinical training at the Clinical Education Center, Ratchaburi Hospital.

### Curriculum

The Doctor of Medicine program at the Faculty of Medicine Siriraj Hospital, Mahidol University, Thailand, spans 6 years, divided into the preclinical years (years 1‐3) and the clinical years (years 4‐6). The latest curriculum, implemented in 2021, introduced several changes, including defining specific CLOs, transferring 4 basic biomedical science courses from year 2 of the 2017 curriculum to year 1 of the 2021 curriculum, changing from compensatory to noncompensatory evaluation, and shifting from grading to nongrading evaluation as Outstanding (“O”), Satisfactory (“S”), or Unsatisfactory (“U”).

### Assessment and Evaluation

For each CLO performance, students were permitted unlimited attempts, except for course 4, which allowed only 1 attempt with an 80% passing threshold. For summative assessment, examinations for 4 courses were conducted simultaneously on the same day with a minimum pass mark of 60% for courses 1‐3 and 50% for course 4. For evaluation, students who failed the CLO assessment or the initial attempt at a summative assessment received an “X” grade, indicating that they had not yet passed. If these students subsequently passed the CLO within the specified time frame and achieved at least a 60% score on their second attempt at the summative assessment, they were awarded an “S” grade, but they forfeited eligibility for an “O” grade. To qualify for an “O” grade, students had to attain summative scores of at least 85% in courses 1, 2, and 4, and 80% in course 3, without receiving any “X” grades in any CLO, attitudinal assessments, or summative assessments. A “U” grade was given to students who failed the remediation of the CLO examination, the second attempt at the summative examination, or the assessment of their attitude.

### Assessment Engagement Statistics

Students’ assessment engagement statistics comprised 319 students: 23 in the academic group, 6 in the quota group, 261 in the test group, and 29 in the rural group. These statistics encompassed various aspects, including the number of total, intentional, and unintentional attempts; instances of first-pass attempt and highest scoring attempt; the number of passings for each CLO; and (an) additional attempts after passing each CLO. Across the 4 courses, course 1 included 5 CLOs, course 2 included 4 CLOs, course 3 included 5 CLOs, and course 4 included 5 CLOs.

For each CLO, the number of total attempts is the total number of times the students accessed the assessment platform. The number of intentional attempts is defined as attempts in which ≥50% of the items were completed. The number of unintentional attempts is defined as attempts in which <50% of the items were completed. The instances of the first-pass attempt are the first instances when the students successfully meet the passing level. The instances of the highest-scoring attempt are the instances when the students achieve the maximum score. The number of passings for each CLO is the count of instances in which the students successfully meet the passing level. The additional attempts after passing each CLO are the number of subsequent instances after students have already passed.

### Questionnaires

The questionnaires were developed collaboratively by the authors under the supervision of the deputy dean for undergraduate education at the Faculty of Medicine Siriraj Hospital. The questionnaires regarding students’ SRL strategies and preferences regarding nongrading evaluation among the academic, quota, test, and rural groups were administered to first-year medical students during the academic year 2021, following the summative examination.

Questionnaire 1, rated on a Likert scale from 1 (strongly disagree) to 5 (strongly agree), assessed students’ perspectives and experiences across four domains: (1) teaching and learning, (2) CLOs and program learning outcomes, (3) nongrading evaluation, and (4) academic obstacles, included to identify barriers perceived by students in adapting to the new structure, learning activities, and nongrading evaluation system.

Questionnaire 1 was initially based on a university-standard template and was subsequently refined using items adapted from 2 well-established instruments: first, the University Students Learning Strategies Questionnaire, which contributed items addressing goal-setting and emotional barriers to learning [23], for example, “I try to set goals before studying,” “I feel discouraged from studying,” and “I have concerns that interfere with my learning.” Second, the Academic Motivation Scale, college version, which informed items related to intrinsic motivation (eg, “I enjoy and feel satisfied when learning new things” and “I feel happy when I achieve each course learning outcome”) and amotivation (eg, “I feel like I waste time participating in class activities”) [24].

Questionnaire 1 represents 3 phases of SRL strategies. The Forethought Phase (P1), representing motivation and beliefs, included items such as goal setting and enthusiasm toward learning and teaching activities. The Performance Phase (P2), reflecting monitoring and strategy use, encompassed concerns or obstacles that hindered studying such as the perceived impact of unlimited CLO assessment attempts. The Self-Reflection Phase (P3), representing assessment and self-reaction, included items related to satisfaction with nongrading evaluation and overall learning experiences gained from the current curriculum. It also explored students’ perceptions of the nongrading “O”/”S”/“U” evaluation, the applicability of knowledge to future clinical and national assessments, and overall satisfaction with the curriculum. Both positively and negatively worded statements were included to support internal consistency testing.

The overall questionnaire 1 response rate was 99.1% (316/319), including 100% (23/23) in the academic group, 100% (6/6) in the quota group, 98.9% (258/261) in the test group, and 100% (29/29) in the rural group. The proportions of respondents were 7.3% (23/316) in the academic group, 1.9% (6/316) in the quota group, 81.6% (258/316) in the test group, and 9.2% (29/316) in the rural group.

Questionnaire 2, which allowed multiple responses, was developed to explore students’ preferences regarding the newly implemented “O”/”S”/“U” grading system, with a particular focus on their perspectives toward the inclusion of the “O” grade, in relation to aspects of SRL strategies (Forethought, Performance, and Self-Reflection phases). While the “O” grade may serve as a form of academic recognition that rewards effort, motivation, and high performance, several student concerns also emerged, particularly regarding its association with extrinsic motivation, heightened anxiety, and increased competitiveness. Although no literature has directly addressed the specific effects of the “O” grade itself, studies on A-F grading suggest that top grades may similarly function as motivators and thus can be reasonably used to approximate the motivational impact of the “O” grade in this context [25]. Based on these recurring themes, the research team identified measurable items that addressed key constructs frequently cited in the literature on grading systems, including stress [9], competition [10,26], study focus [26], effort, and life-goal setting.

Although this questionnaire did not undergo a full validation process and did not adopt an existing standardized instrument, its content was informed by student feedback gathered during monthly online visits by the deputy dean and assistant dean (the corresponding author, CS) of undergraduate education with the first-year cohort, internal faculty discussions, and relevant literature on the psychological and behavioral impacts of grading practices. A qualitatively informed survey design approach was used to generate a focused list of 5 questions designed to capture concrete student perspectives, thereby enabling clearer interpretation of their positions on key issues as part of the validity argument. A simplified yes/no response format was deliberately selected to enhance clarity, improve response process validity, and encourage full participation by prompting students to take a definitive stance on each item.

The overall response rate was 93.7% (299/319), with participation rates of 95.7% (22/23) in the academic group, 100% (6/6) in the quota group, 93.1% (243/261) in the test group, and 96.6% (28/29) in the rural group. The proportions of respondents were 7.4% (22/299) in the academic group, 2.0% (6/299) in the quota group, 81.3% (243/299) in the test group, and 9.4% (28/299) in the rural group.

To ensure content validity, both questionnaires were reviewed by 3 medical education experts. Their feedback was incorporated to improve item clarity, coverage, and logical flow. The finalized questionnaire was administered at the end of the academic year but before grade announcement to minimize potential bias.

Construct validity for questionnaire 1 was examined using exploratory factor analysis (principal component extraction with Varimax rotation). Sampling adequacy was acceptable (Kaiser-Meyer-Olkin Measure of Sampling Adequacy=0.812), and Bartlett’s test of sphericity was significant (*χ*²_136_=2253.45; *P*<.001). Four factors were extracted, explaining 62.9% of the total variance after rotation. Factor loadings supported a theoretically consistent structure aligned with SRL domains: (1) teaching and learning, (2) CLOs and program learning outcomes, (3) nongrading evaluation, and (4) academic obstacles. Most communalities were ≥0.50. Internal consistency was acceptable (Cronbach α=0.729). Content validity was ensured through expert review and adaptation from validated instruments.

For questionnaire 2, the first item assessed grading system preference and was analyzed descriptively because it represents a categorical choice rather than a latent construct. The remaining 5 dichotomous items assessed perceived consequences of the “O” grade (stress, competition, focus, effort, and goals). When treated as a single composite, internal consistency was low (Cronbach α=0.349), indicating that these items did not form a unidimensional scale. Interitem correlations suggested 2 distinct clusters—perceived costs and perceived benefits—with negative correlations between clusters. Given this multidimensional structure and low reliability, items were analyzed individually. Construct validity was not established for questionnaire 2; however, content validity was supported through expert review and literature-informed item development.

### Academic Performance

Academic performance was represented by CLO scores and summative scores, both comprising a total of 319 students: 23 in the academic group, 6 in the quota group, 261 in the test group, and 29 in the rural group.

### Missing Data Handling

Missing data were assessed using the SPSS (version 30; IBM Corp) Missing Value Analysis module. The dataset for assessment engagement statistics and CLO scores included 319 students, and summative scores were available for all 319 students. No missing data were observed for questionnaire 1 (N=316) and questionnaire 2 (N=299), as well as for summative scores. For assessment engagement statistics and CLO scores, a small proportion of missing data occurred exclusively in the test group due to some students not attempting specific CLO assessments or data unavailability. Specifically, missing data were observed for CLO 1 (3 students), CLO 2 (5 students), CLO 3 (3 students), CLO 4 (6 students), and CLO 5 (4 students) in course 1; for CLO 1 (3 students), CLO 2 (3 students), CLO 3 (6 students), and CLO 4 (7 students) in course 2; and for CLOs 1‐5 (3 students) in course 3. Across all CLO variables, the proportion of missing data ranged from 0.94% to 2.19%, which is below the commonly accepted threshold of 5%.

Little’s Missing Completely at Random (MCAR) test was performed on the combined dataset of assessment engagement statistics and CLO scores using the SPSS Missing Value Analysis module. The test was nonsignificant (*χ*²_464_=0.000; *P*=1.000), indicating that the observed missing data pattern was consistent with MCAR. Because the proportion of missing data was very small and the MCAR assumption was satisfied, complete case analysis was applied. Multiple imputation was not performed.

### Reporting Guidelines

To enhance transparency and clarity of reporting, this study adhered to EQUATOR-aligned guidelines appropriate to its methodological structure. The STROBE (Strengthening the Reporting of Observational Studies in Epidemiology) guideline was applied to the retrospective observational components [27], and the GRAMMS (Good Reporting of A Mixed Methods Study) guideline was applied to the mixed methods framework [28]. Completed STROBE and GRAMMS checklists have been uploaded as supplementary files.

### Statistics

For statistical analysis, SPSS (version 18) was used. Participant demographics and students’ preferences regarding nongrading evaluation are presented as frequencies (N) and percentages. Students’ assessment engagement statistics are reported as ranges of means and medians. Comparisons of SRL, assessment engagement statistics, and CLO and summative scores among students in the academic, quota, test, and rural groups were analyzed using one-way ANOVA and are expressed as means with 95% CIs. Post hoc analyses using Fisher least significant difference test were performed, as appropriate. The correlations of summative scores with students’ assessment engagement statistics and CLO scores within the academic, quota, test, and rural groups were analyzed using Pearson correlation coefficient (*R*) with 95% CIs. Multiple linear regression analyses were conducted to identify factors significantly associated with summative scores in these groups, with regression coefficients reported alongside their 95% CIs. Statistical significance was set at *P*<.05.

## Results

### Participant Demographics by Admission Pathway

The demographic characteristics of participants across the 4 TCAS admission groups—academic, quota, test, and rural—demonstrated distinct patterns in age, sex, school region, and hometown region, as shown in Table 1. Most students were aged between 18 and 20 years, except in the quota group, where one-third were aged 21‐25 years and another one-third 26‐30 years. Accordingly, the mean age was highest in the quota group (23.7 years), compared with 19.4‐19.5 years in the other groups. Male students predominated in the academic (15/23, 65.2%), test (134/261, 51.3%), and rural (17/29, 58.6%) groups, whereas the quota group was primarily female (5/6, 83.3%). By school region, Bangkok was most common in the test group (147/261, 56.3%), the central region predominated in the academic group (15/23, 65.2%), and the western region was most frequent in the rural group (22/29, 75.9%). The quota group showed a more even distribution, with 50.0% (3/6) from the central region and 16.7% (1/6) each from the west, south, and Bangkok. By hometown region, Bangkok accounted for the largest share in the test group (122/261, 46.7%); the academic group was distributed across Bangkok (9/23, 39.1%), central (5/23, 21.7%), and northeast (5/23, 21.7%); and the rural group primarily originated from the western region (23/29, 79.3%). The quota group consisted of one-third each from Bangkok and the central region, with smaller proportions from the west and south (1/6, 16.7% each).

**Table 1.** Participant demographics by admission pathway.

Factors	Academic group (N=23), n (%)		Quota group (N=6), n (%)		Test group (N=261), n (%)		Rural group (N=29), n (%)	
Age (years)								
18‐20	23 (100.0)		2 (33.4)		246 (94.2)		29 (100.0)	
21‐25	N/A^a^		2 (33.3)		15 (5.8)		N/A	
26‐30	N/A		2 (33.4)		N/A		N/A	
Mean	19.4		23.7		19.5		19.5	
Median	19.4		24.0		19.4		19.5	
Range	18.7‐20.0		19.4‐29.7		18.0‐22.7		18.8‐20.0	
Sex								
Male	15 (65.2)		1 (16.7)		134 (51.3)		17 (58.6)	
Female	8 (34.8)		5 (83.3)		127 (48.7)		12 (41.4)	
School region								
Bangkok	3 (13.0)		1 (16.7)		147 (56.3)		N/A	
Central	15 (65.2)		3 (50.0)		90 (34.5)		7 (24.1)	
North	1 (4.4)		N/A		2 (0.8)		N/A	
Northeast	3 (13.0)		N/A		9 (3.5)		N/A	
East	1 (4.4)		N/A		3 (1.1)		N/A	
West	N/A		1 (16.7)		N/A		22 (75.9)	
South	N/A		1 (16.7)		10 (3.8)		N/A	
Hometown region								
Bangkok	9 (39.1)		2 (33.3)		122 (46.7)		N/A	
Central	5 (21.7)		2 (33.3)		59 (22.6)		6 (20.7)	
North	1 (4.4)		N/A		3 (1.2)		N/A	
Northeast	5 (21.7)		N/A		24 (9.2)		N/A	
East	2 (8.7)		N/A		11 (4.2)		N/A	
West	N/A		1 (16.7)		8 (3.1)		23 (79.3)	
South	1 (4.4)		1 (16.7)		34 (13.0)		N/A	

a N/A: not applicable.

### Comparisons of SRL Strategies Among Academic, Quota, Test, and Rural Groups

Comparisons of SRL strategies among academic, quota, test, and rural groups are shown in Table 2. In the aspect of teaching and learning, students in the academic group reported significantly higher levels of actively setting goals before studying than those in the quota group (*P*=.044; Table 2). The 95% CI for the academic group was narrow (4.07‐4.63), indicating high precision. In contrast, the quota group showed a much wider 95% CI (2.58‐4.75), reflecting lower precision due to both the small sample size and the high variability in students’ responses within this group (Table 2). Students in the academic group also reported significantly higher levels of feeling enthusiastic about learning and teaching activities than students in the rural group (*P*=.042; Table 2). The 95% CI for the academic group (4.18‐4.69) was narrow, suggesting stable estimates, whereas the rural group’s moderately wide 95% CI (3.65‐4.28) indicates greater variability in enthusiasm levels among students in this pathway (Table 2).

**Table 2.** Comparisons of self-regulated learning strategies among academic, quota, test, and rural groups.

Questions		Academic group (N=23)	Quota group (N=6)	Test group (N=258)	Rural group (N=29)
		Mean (95% CI)	Mean (95% CI)	Mean (95% CI)	Mean (95% CI)
Teaching and learning					
1	I actively set goals for myself before studying. (P1^a^)	4.35 (4.07‐4.63)	3.67 (2.58-4.75)^b^	4.22 (4.13‐4.31)	4.10 (3.79‐4.41)
2	I am enthusiastic about learning and teaching activities. (P1^a^)	4.43 (4.18‐4.69)	3.83 (2.80‐4.87)	4.12 (4.02‐4.22)	3.97 (3.65–4.28)^b^
3	I am happy and satisfied when I learn something new. (P^3c^)	4.39 (4.08‐4.70)	4.50 (3.93‐5.07)	4.41 (4.32‐4.50)	4.34 (4.11‐4.58)
4	The teaching and learning management of this curriculum helps me experience learning in an excellent manner. (P3^c^)	4.39 (4.05‐4.73)	4.67 (4.12‐5.21)	4.38 (4.29‐4.46)	4.31 (4.10‐4.52)
5	Overall, I am satisfied with the learning experience gained from the current curriculum. (P3^c^)	4.35 (4.14‐4.56)	4.67 (4.12‐5.21)	4.35 (4.27‐4.43)	4.38 (4.12‐4.64)
CLOs^d^ and PLOs^e^					
6	I feel happy when I achieve the CLOs each time. (P3^c^)	4.83 (4.66‐4.99)	5.00 (5.00‐5.00)	4.62 (4.55‐4.70)	4.55 (4.36‐4.74)
7	I like the opportunity to have unlimited attempts at achieving the CLOs. (P2^f^)	4.74 (4.54‐4.93)	4.83 (4.40‐5.26)	4.85 (4.80‐4.90)	4.76 (4.56‐4.95)
8	I think that the knowledge and experience gained from studying in the first year can be applied in future medical studies. (P3^c^)	4.61 (4.36‐4.86)	5.00 (5.00‐5.00)	4.67 (4.61‐4.74)	4.72 (4.55‐4.90)
9	I think that the knowledge and experience gained from studying will be applicable to the preclinical comprehensive examination and the National License Examination step 1. (P3^c^)	4.74 (4.54‐4.93)	4.83 (4.40‐5.26)	4.64 (4.57‐4.71)	4.62 (4.41‐4.83)
10	The current examination method (once/semester) allows me to study happily. (P3^c^)	3.17 (2.64‐3.71)	3.33 (2.48‐4.19)	3.40 (3.24‐3.56)	3.24 (2.81‐3.67)
Nongrading evaluation					
11	I am happy to learn through the nongrading evaluation (“O”/”S”/“U”). (P3^c^)	4.35 (4.07‐4.63)	5.00 (5.00–5.00)^b^	4.77 (4.70‐4.84)^g^	4.86 (4.73‐5.00)^g^
12	I agree with changing from the grading to the nongrading evaluation (“O”/”S”/“U”) for all preclinical courses. (P3^c^)	4.35 (4.07‐4.63)	5.00 (5.00–5.00)^b^	4.79 (4.72‐4.86)^h^	4.90 (4.78‐5.01)^h^
Academic obstacles					
13	I feel like I’m wasting my time participating in the teaching and learning activities. (P2^f^)	1.83 (1.47‐2.19)	1.67 (0.81‐2.52)	2.07 (1.91‐2.22)	2.00 (1.55‐2.45)
14	I have concerns or problems that prevent me from studying. (P2^f^)	2.13 (1.60‐2.66)	1.83 (1.04‐2.62)	2.60 (2.45‐2.76)	2.76 (2.38‐3.13)
15	I don’t feel like studying. (P1^a^)	1.87 (1.43‐2.31)	2.00 (1.06‐2.94)	2.37 (2.22‐2.52)	2.38 (1.98‐2.78)
16	I have stress when reading books or following lessons during studies. (P2^f^)	2.64 (2.15‐3.12)	3.00 (1.67‐4.33)	3.27 (3.14‐3.40)^g^	3.52 (3.20‐3.83)^g^
17	I have stress when reading books before exams. (P2^f^)	3.70 (3.11‐4.29)^i^	3.50 (1.78‐5.22)	3.97 (3.85‐4.10)^i^	4.38 (4.10‐4.66)

a P1 represents Motivation and Beliefs (motivation and beliefs (Forethought Phase).

b *P*<.05 compared with the academic group.

c P3 represents assessment and self-reaction (Self-Reflection Phase).

d CLOs: course learning outcomes.

e PLOs: program learning outcomes.

f P2 represents Monitoring and Strategy Use (Performance Phase).

g *P*<.01 compared with the academic group.

h *P*<.001 compared with the academic group.

i *P*<.05 compared with the rural group.

In the aspect of nongrading evaluation, students in the academic group reported significantly lower levels of agreement with learning via nongrading evaluation (“O”/“S”/“U”) than the quota group (*P*=.012), test group (*P*=.001), and rural group (*P*=.001) (Table 2). The 95% CI for the academic group (4.07‐4.63) was relatively narrow, indicating high precision and consistent responses, whereas the quota group showed a ceiling effect (5.00‐5.00) suggesting no variability, and the 95% CIs for the test (4.70‐4.84) and rural groups (4.73‐5.00) were very narrow, reflecting precise estimates (Table 2). Similarly, students in the academic group reported significantly lower agreement with changing from grading to nongrading evaluation (“O”/“S”/“U”) for all preclinical courses than the quota group (*P*=.011), test group (*P*<.001), and rural group (*P*<.001; Table 2). The 95% CI for the academic group (4.07‐4.63) was narrow, suggesting stable estimates, whereas the quota group again showed a ceiling effect (5.00‐5.00), and the test (4.72‐4.86) and rural groups (4.78‐5.01) had narrow 95% CIs, indicating high precision (Table 2).

In the aspect of academic obstacles, students in the academic group rated significantly lower levels of stress when reading books or following lessons during studies than students in the test (*P*=.007) and rural groups (*P*=.003; Table 2). The 95% CI for the academic group (2.15‐3.12) was moderately wide, indicating greater variability in perceived stress, whereas the test (3.14‐3.40) and rural groups (3.20‐3.83) showed narrower 95% CIs reflecting more consistent responses, and the quota group demonstrated a wide 95% CI (1.67‐4.33), indicating low precision due to both high variability and a small sample size (Table 2).

Furthermore, students in the rural group rated significantly higher levels of stress when reading books before examinations than students in the academic (*P*=.02) and test groups (*P*=.046; Table 2). The 95% CI for the rural group (4.10‐4.66) was relatively narrow, indicating high precision, while the 95% CI for the academic group (3.11‐4.29) was moderately wide, suggesting moderate precision, and the 95% CI for the test group (3.85‐4.10) was narrow, reflecting precise estimates; the quota group had an extremely wide 95% CI (1.78‐5.22), indicating high variability and small sample size (Table 2).

### Students’ Preferences Regarding Nongrading Evaluation

Students’ preferences regarding nongrading evaluation and the “O” grade in academic, quota, test, and rural groups are shown in Table 3. Students in the academic, quota, and test groups predominately selected “O”/“S”/“U” for 63.6% (14/22), 83.3% (5/6), and 54.3% (132/243), respectively, while students in the rural group mainly selected “S”/“U” at 46.4% (13/28; Table 3). Furthermore, students in the academic and quota groups reported that “O” mainly made them more focused on studying and led to increased effort with equal percentage (15/22, 68.2% and 3/6, 50%, respectively; Table 3). In addition, students in the test and rural groups reported that “O” mostly led to increased effort (161/243, 66.3% and 18/28, 64.3%, respectively; Table 3).

**Table 3.** Students’ preferences regarding nongrading evaluation.

	Academic group (N^a^=22), n (%)		Quota group (N=6), n (%)		Test group (N=243), n (%)		Rural group (N=28), n (%)	
Choosing the evaluation								
“S”^a^/“U”^b^	7 (31.8)		1 (16.7)		90 (37.0)		13 (46.4)	
“O”^c^/“S”/“U”	14 (63.6)		5 (83.3)		132 (54.3)		12 (42.9)	
Both “S”/“U” and “O”/“S”/“U”	1 (4.6)		N/A^d^		21 (8.7)		3 (10.7)	
Topics								
“O” makes students feel stressed. (P3^e^)	10 (45.5)		N/A		83 (34.2)		11 (39.3)	
“O” leads to increased competition. (P2^f^)	13 (59.1)		1 (16.7)		98 (40.3)		11 (39.3)	
“O” makes students more focused on studying. (P2)	15 (68.2)		3 (50.0)		116 (47.7)		12 (42.9)	
“O” leads to increased effort. (P2)	15 (68.2)		3 (50.0)		161 (66.3)		18 (64.3)	
“O” leads to having more goals in life. (P1^g^)	13 (59.5)		1 (16.7)		104 (42.8)		13 (46.4)	

a “S”: satisfactory.

b “U”: unsatisfactory.

c “O”: outstanding.

d N/A: not applicable.

e P3 represents assessment and self-reaction (Self-Reflection Phase).

f P2 represents monitoring and strategy use (Performance Phase).

g P1 represents motivation and beliefs (Forethought Phase).

### Students’ Assessment Engagement Statistics in Performing Each CLO

Students’ assessment engagement statistics in performing each CLO of students in academic, quota, test, and rural groups are shown as ranges of means and medians in Table 4. These assessment engagement statistics include multiple aspects of assessment engagement, namely, total attempts, intentional and unintentional attempts, first-pass attempts, highest scoring attempts, passing instances, and additional attempts after passing for each CLO.

**Table 4.** Students’ assessment engagement statistics in performing each course learning outcome.

Factors	Academic group (N=23)		Quota group (N=6)		Test group (N=258)		Rural group (N=29)	
	Mean (range)	Median (range)	Mean (range)	Median (range)	Mean (range)	Median (range)	Mean (range)	Median (range)
Number of total attempts of each CLO^a^.	2.0‐9.1	1‐7	3.5‐29.0	2.5‐19	2.6‐15.3	2‐8	2.4‐12.3	2‐10
Number of intentional attempts of each CLO.	1.9‐6.3	1‐5	2.0‐6.3	2‐5.5	2.0‐7.5	2‐6	2.3‐9.8	2‐9
Number of unintentional attempts of each CLO.	0‐5.1	0	0‐22.8	0‐8.5	0‐10.9	0	0‐6.5	0
Instances of first-pass attempt of each CLO.	1.4‐3.6	1‐3	1.3‐4.0	1‐3	1.4‐4.5	1‐3	1.9‐6.5	1‐5
Instances of highest scoring attempt of each CLO.	1.7‐5.7	1‐4	1.7‐6.0	1.5‐5	1.9‐6.7	2‐5	2.2‐9.5	2‐9
Number of passings for each CLO.	1.5‐3.0	1‐2	1.5‐4.0	1‐3.5	1.4‐3.3	1‐2	1.4‐2.9	1‐2
Additional attempts after passing each CLO.	0.5‐2.7	0‐2	0.5‐4.0	0‐3	0.6‐3.0	0‐1	0.4‐3.4	0‐1

a CLO: course learning outcome.

### Comparisons of Assessment Engagement Statistics Among Students in Academic, Quota, Test, and Rural Groups

**Figure 1.**
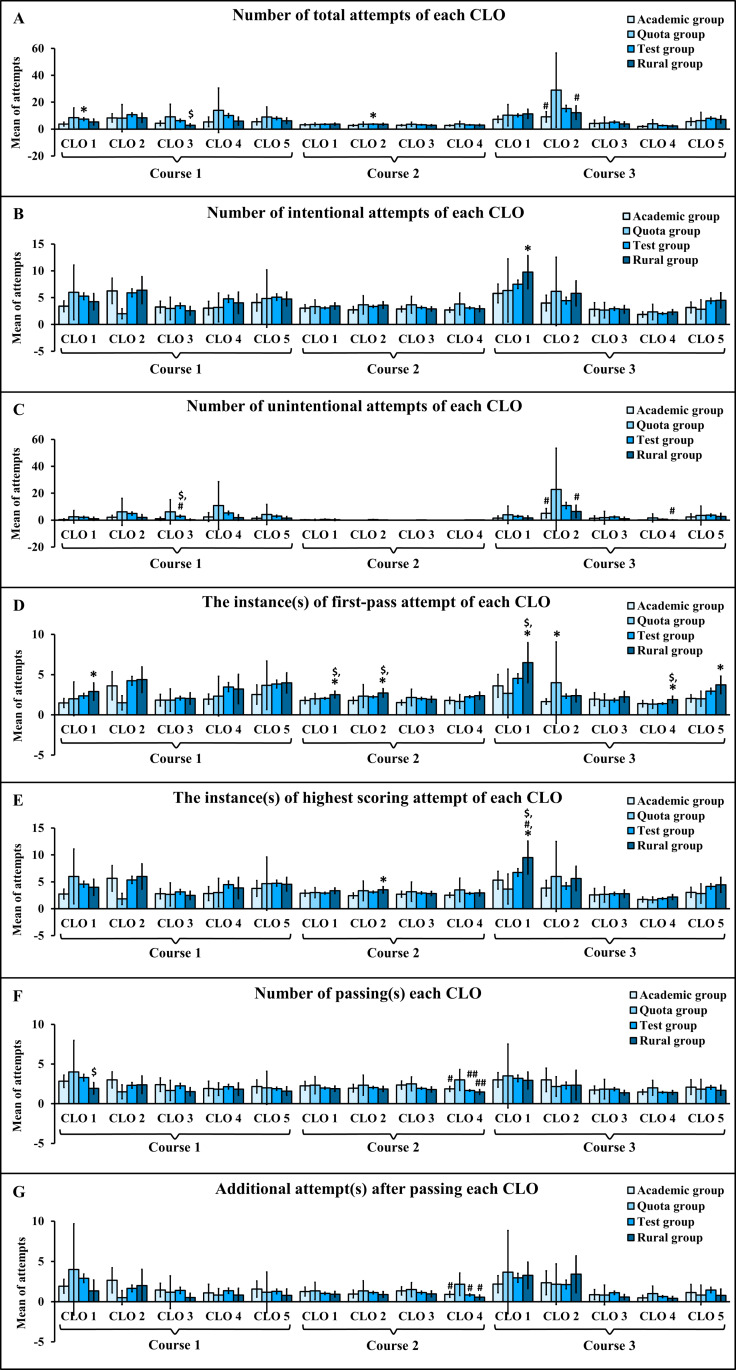
Comparisons of assessment engagement statistics among students in academic, quota, test, and rural groups. Data are presented as mean with 95% CIs. *P<.05 compared with students in the academic group; ^#^*P*<.05, ^##^*P*<.001 compared with students in the quota group; and ^$^*P*<.05 compared with students in the test group. (A) Number of total attempts of each CLO. (B) Number of intentional attempts of each CLO. (C) Number of unintentional attempts of each CLO. (D) The instance(s) of first-pass attempt of each CLO. (E) The instance(s) of highest scoring attempt of each CLO. (F) Number of passing(s) each CLO. (G) Additional attempt(s) after passing each CLO. CLO: course learning outcome.

Comparisons of assessment engagement statistics among students in academic, quota, test, and rural groups are shown in Figure 1. For each CLO, the number of total attempts was significantly higher in the test group compared with the academic group in course 1 CLO 1 (*P*=.044) and course 2 CLO 2 (*P*=.049) and compared with the rural group in course 1 CLO 3 (*P*=.03), as well as significantly higher in the quota group compared with the academic (*P*=.02) and rural groups (*P*=.041) in course 3 CLO 2 (Figure 1A). The number of intentional attempts was significantly lower in the academic group in course 3 CLO 1 compared with the rural group (*P*=.027; Figure 1B). The number of unintentional attempts was significantly higher in the quota group compared with the rural group in course 1 CLO 3 (*P*=.045), course 3 CLO 2 (*P*=.043), and course 4 (*P*=.048), and compared with the academic group in course 3 CLO 2 (*P*=.03), as well as significantly higher in the test group compared with the rural group in course 1 CLO 3 (*P*=.042; Figure 1C). The instances of first-pass attempt were significantly lower in the academic group compared with the rural group in course 1 CLO 1 (*P*=.04), course 2 CLO 1 (*P*=.008) and 2 (*P*=.004), course 3 CLO 1 (*P*=.03), CLO 4 (*P*=.044), and CLO 5 (*P*=.03), and compared with the quota group in course 3 CLO 2 (*P*=.02), as well as significantly lower in the test group compared with the rural group in course 2 CLO 1 (*P*=.02) and CLO 2 (*P*=.03) and course 3 CLO 1 (*P*=.04) and 4 (*P*=.005) (Figure 1D). The instances of highest scoring attempts were significantly higher in the rural group compared with the academic group in course 2 CLO 2 (*P*=.02) and compared with the academic group (*P*=.01), quota group (*P*=.03), and test group (*P*=.02) in course 3 CLO 1 (Figure 1E). The number of passings in each CLO was significantly lower in the rural group compared with the test group in course 1 CLO 1 (*P*=.03), as well as significantly higher in the quota group compared with the academic group (*P*=.004), test group (*P*<.001), and rural group (*P*<.001) in course 2 CLO 4 (Figure 1F). The additional attempts after passing each CLO were significantly higher in the quota group compared with the academic group (*P*=.01), test group (*P*=.004), and rural group (*P*=.001) in course 2 CLO 4 (Figure 1G).

Across total attempts (Figure 1A) and unintentional attempts (Figure 1C), the test group consistently demonstrated narrow 95% CIs indicating high precision, the academic and rural groups showed 95% CIs of narrow to moderate width indicating high to moderate precision, and the quota group exhibited extremely wide 95% CIs reflecting low precision due to substantial variability and a small sample size.

Across intentional attempts (Figure 1B), first-pass attempts (Figure 1D), highest scoring attempts (Figure 1E), number of passings (Figure 1F), and additional attempts after passing (Figure 1G), a similar precision pattern was observed, in which the test group consistently showed narrow 95% CIs indicating high precision, the academic and rural groups demonstrated moderate width 95% CIs reflecting moderate precision, and the quota group exhibited the widest 95% CIs indicating low precision due to high variability and its small sample size.

### Comparisons of CLO and Summative Scores Among Students in Academic, Quota, Test, and Rural Groups

**Figure 2.**
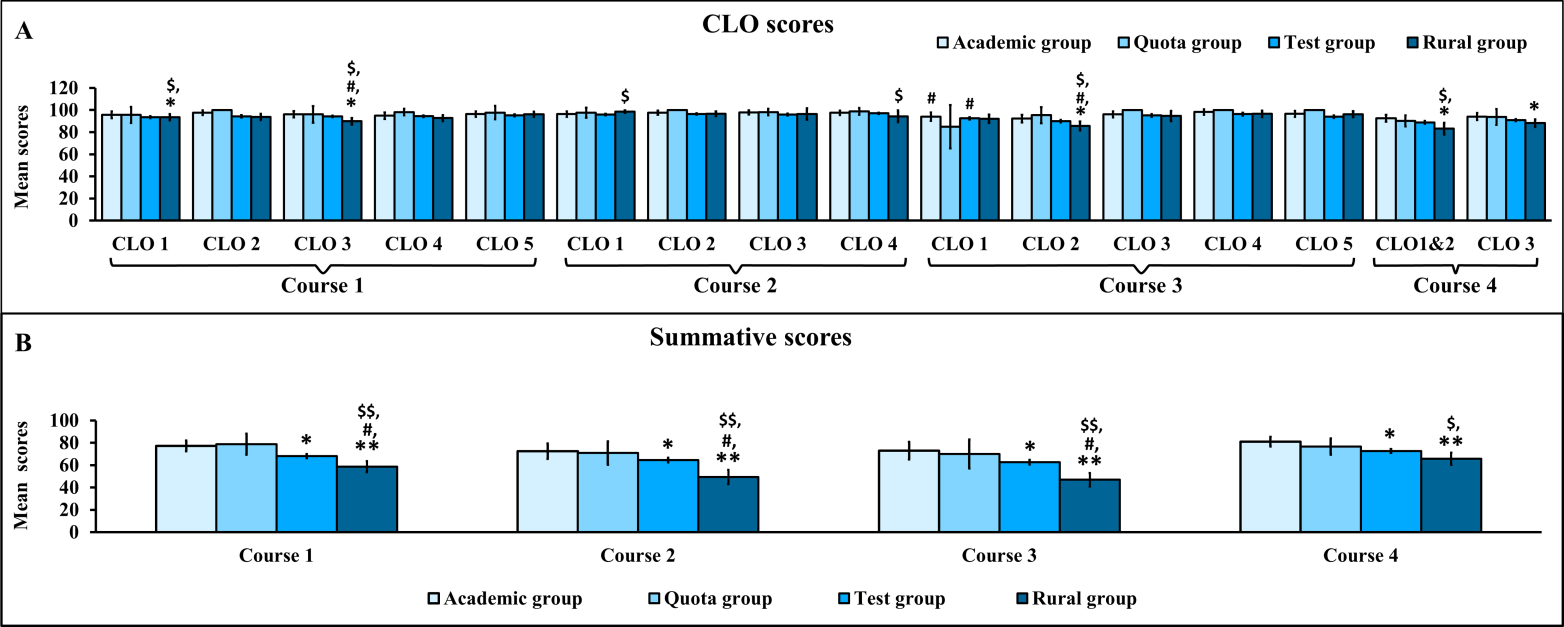
Comparisons of CLO and summative scores among students in academic, quota, test, and rural groups. Data are presented as mean with 95% CIs. *P<.05, **P<.001 compared with students in the academic group; ^#^*P*<.05 compared with students in the quota group; and ^$^*P*<.05, ^$$^*P*<.001 compared with students in the test group. (A) CLO scores. (B) Summative scores. CLO: course learning outcome.

Comparisons of CLO and summative scores among students in academic, quota, test, and rural groups are shown in Figure 2.

Some CLO scores (Figure 2A) and all summative scores (Figure 2B) in courses 1‐4 were significantly lower in the rural group when compared with the academic, quota, and test groups (*P*<.05 all). Across CLO scores (Figure 2A) and summative scores (Figure 2B), a similar precision pattern was observed, in which the test group consistently showed narrow 95% CIs indicating high precision, the academic and rural groups demonstrated moderate-width 95% CIs reflecting moderate precision, and the quota group exhibited the widest 95% CIs indicating low precision due to substantial variability and a small sample size.

### Correlations of Summative Scores With Students’ Assessment Engagement Statistics and CLO Scores Within the Course

Correlations of summative scores with students’ assessment engagement statistics and CLO scores within the course of students in academic, quota, test, and rural groups are shown in Multimedia Appendix 1.

In the academic group, summative scores of each course were positively correlated with number of passings each CLO of courses 1 and 3 (*R*=0.415‐0.580), additional attempts after passing each CLO of course 1 (*R*=0.415‐0.555), and CLO scores of all courses (*R*=0.432‐0.707) but were negatively correlated with number of total attempts (*R*=−0.450) and number of unintentional attempts (*R*=−0.511) of course 1 and instances of first-pass attempt of courses 1 and 2 (*R*=(−0.437) – (−0.491)) (*P*<.05 all; Multimedia Appendix 1).

In the quota group, summative scores of each course exhibited positive correlation with number of total attempts (*R*=0.814) and number of intentional attempts (*R*=0.926) of course 2 and CLO scores of course 3 (*R*=0.844) but had negative correlations with number of total attempts (*R*=−0.964) and number of unintentional attempts (*R*=−0.845) of course 3 (*P*<.05 all; Multimedia Appendix 1).

In the test group, summative scores of each course were significantly positively correlated with number of passings each CLO (*R*=0.164‐0.233) and additional attempts after passing each CLO (*R*=0.125‐0.209) of courses 1, 2, and 3; CLO scores of all courses (*R*=0.129‐0.253); and number of intentional attempts of course 3 (*R*=0.177) but were significantly negatively correlated with number of total attempts (*R*=(−0.128) – (−0.233)) and number of unintentional attempts (*R*=(−0.148) –(−0.208)) of courses 1 and 3; number of intentional attempts of course 1 (*R*=−0.134); instances of first-pass attempt of courses 1, 2, and 3 (*R*=(−0.123) – (−0.344)); and instances of highest scoring attempt of courses 1 and 2 (*R*=(−0.153) – (−0.160)) (*P*<.05 all; Multimedia Appendix 1).

In the rural group, summative scores of each course showed positive correlations with CLO scores of courses 1, 3, and 4 (*R*=0.420‐0.725) and number of unintentional attempts (*R*=0.393) and number of passings each CLO (*R*=0.390) of course 3 but exhibited negative correlations with number of intentional attempts of courses 2 and 3 (*R*=(−0.399)–(−0.577)); instances of first-pass attempt of courses 2 and 3 (*R*=(−0.388) – (−0.655); and instances of highest scoring attempt of courses 2 and 3 (*R*=(−0.441) – (−0.614)) (*P*<.05 all; Multimedia Appendix 1).

Notably, the scores for course 1 CLO 2, course 2 CLO 2, and course 3 CLO 3, 4, and 5 in the quota group did not correlate with the summative scores because all students in this group received a score of 100 for these CLOs. Across correlations between summative scores and assessment engagement statistics and CLO scores, a similar precision pattern was observed, in which the test group consistently showed narrow 95% CIs indicating high precision, the academic and rural groups demonstrated moderate-width 95% CIs reflecting moderate precision, and the quota group exhibited the widest 95% CIs indicating low precision due to substantial variability and a small sample size (Multimedia Appendix 1).

### Multiple Linear Regression Analyses of Summative Scores

Multiple linear regression analyses of summative scores in academic, quota, test, and rural groups are shown in Table 5.

**Table 5.** Multiple linear regression analyses of summative scores.

Model	*R*	*R* ^2^	*P* value		Coefficient	SE	*t* test (*df*)	*P* value	95% CI
Academic group									
Summative scores of course 1									
1	0.630	0.397	.001^a^						
				Constant	−40.301	31.689	−1.272 (21)	.217	−106.203 to 25.600
				CLO5^b^ scores	1.221	0.329	3.717 (21)	.001^a^	0.538 to 1.905
2	0.722	0.521	<.001^c^						
				Constant	−17.197	30.682	−0.560 (20)	.581	−81.198 to 46.804
				CLO5 scores	0.912	0.330	2.769 (20)	.012^d^	0.225 to 1.600
				Number of passings (CLO1)	2.349	1.033	2.273 (20)	.034^d^	0.193 to 4.504
3	0.784	0.614	<.001^c^						
				Constant	−1.143	29.210	−0.039 (19)	.969	−62.280 to 59.994
				CLO5 scores	0.764	0.311	2.456 (19)	.024^d^	0.113 to 1.415
				Number of passings (CLO1)	2.112	0.957	2.206 (19)	.040^d^	0.108 to 4.116
				Number of unintentional attempts (CLO4)	−0.466	0.217	−2.148 (19)	.045^d^	−0.920 to −0.012
Summative scores of course 2									
1	0.491	0.241	.017^d^						
				Constant	88.704	6.934	12.793 (21)	<.001^c^	74.284 to 103.124
				Instances of first-pass attempt (CLO3)	−10.648	4.125	−2.582 (21)	.017^d^	−19.226 to −2.070
Summative scores of course 3									
1	0.707	0.500	<.001^c^						
				Constant	−119.198	42.038	−2.835 (21)	.010^d^	−206.621 to (−31.776)
				CLO5 scores	1.991	0.435	4.580 (21)	<.001^c^	1.087 to 2.894
2	0.778	0.606	<.001^c^						
				Constant	−160.196	42.113	−3.804 (20)	.001^a^	−248.043to −72.350
				CLO5 scores	2.285	0.415	5.504 (20)	<.001^c^	1.419 to 3.150
				Number of passings (CLO4)	8.538	3.678	2.322 (20)	.031^d^	0.866 to 16.209
Summative scores of course 4									
1	0.520	0.271	.011^d^						
				Constant	16.399	23.234	0.706 (21)	.488	−31.920 to 64.717
				CLO1 and 2 scores	0.699	0.250	2.793 (21)	.011^d^	0.178 to 1.219
Quota group									
Summative scores of course 2									
1	0.926	0.857	.008^a^						
				Constant	45.455	5.454	8.335 (4)	.001^a^	30.313 to 60.596
				Number of intentional attempts (CLO1)	7.614	1.553	4.903 (4)	.008^a^	3.302 to 11.925
Summative scores of course 3									
1	0.964	0.929	.002^a^						
				Constant	86.429	2.719	31.782 (4)	<.001^c^	78.878 to 93.979
				Number of total attempts (CLO4)	−4.107	0.567	-7.243 (4)	.002^d^	−5.681 to −2.533
Test group									
Summative scores of course 1									
1	0.344	0.118	<.001^c^						
				Constant	73.796	1.251	58.999 (256)	<.001^c^	71.333 to 76.259
				Instances of first-pass attempt (CLO3)	−2.653	0.453	−5.856 (256)	<.001^c^	−3.545 to −1.761
2	0.399	0.159	<.001^c^						
				Constant	70.442	1.546	45.554 (254)	<.001^c^	67.396 to 73.487
				Instances of first-pass attempt (CLO3)	−2.649	0.444	−5.963 (254)	<.001^c^	−3.523 to −1.774
				Number of passing (CLO5)	1.765	0.498	3.545 (254)	<.001^c^	0.784 to 2.745
3	0.433	0.187	<.001^c^						
				Constant	71.500	1.565	45.689 (253)	<.001^c^	68.418 to 74.582
				Instances of first-pass attempt (CLO3)	−2.558	0.439	−5.833 (253)	<.001^c^	−3.422 to −1.695
				Number of passing (CLO5)	1.638	0.492	3.327 (253)	.001^a^	0.668 to 2.608
				Number of unintentional attempts (CLO5)	−0.344	0.116	−2.953 (253)	.003^a^	−0.573 to −0.115
Summative scores of course 2									
1	0.260	0.068	<.001^c^						
				Constant	71.040	1.797	39.530 (253)	<.001^c^	67.501 to 74.580
				Instances of first-pass attempt (CLO3)	−3.164	0.739	−4.282 (253)	<.001^c^	−4.620 to −1.709
2	0.370	0.137	<.001^c^						
				Constant	16.729	12.185	1.373 (252)	.171	−7.269 to 40.727
				Instances of first-pass attempt (CLO3)	−3.289	0.713	-4.614 (252)	<.001^c^	−4.693 to −1.885
				CLO3 scores	0.568	0.126	4.503 (252)	<.001^c^	0.320 to 0.817
3	0.402	0.162	<.001^c^						
				Constant	21.178	12.145	1.744 (251)	.082	−2.742 to 45.098
				Instances of first-pass attempt (CLO3)	−3.222	0.704	−4.574 (251)	<.001^c^	−4.610 to −1.835
				CLO3 scores	0.472	0.130	3.643 (251)	<.001^c^	0.217 to 0.727
				Number of passing (CLO2)	2.260	0.833	2.714 (251)	.007^a^	0.620 to 3.900
4	0.420	0.176	<.001^c^						
				Constant	24.308	12.157	2.000 (250)	.047^d^	0.366 to 48.251
				Instances of first-pass attempt (CLO3)	−2.881	0.719	−4.009 (250)	<.001^c^	−4.296 to −1.465
				CLO3 scores	0.455	0.129	3.525 (250)	.001^a^	0.201 to 0.709
				Number of passing (CLO2)	3.663	1.065	3.441 (250)	.001^a^	1.566 to 5.760
				Instances of highest scoring attempts (CLO2)	−1.625	0.777	−2.093 (250)	.037^d^	−3.155 to −0.096
Summative scores of course 3									
1	0.211	0.045	<.001^c^						
				Constant	59.682	1.385	43.099 (256)	<.001^c^	56.955 to 62.409
				Number of passing (CLO1)	1.032	0.299	3.456 (256)	.001^a^	0.444 to 1.621
2	0.310	0.096	<.001^c^						
				Constant	63.643	1.701	37.406 (255)	<.001^c^	60.292 to 66.994
				Number of passing (CLO1)	1.014	0.291	3.483 (255)	.001^a^	0.441 to 1.587
				Instances of first-pass attempt (CLO3)	−2.142	0.560	−3.822 (255)	<.001^c^	−3.246 to −1.038
3	0.354	0.125	<.001^c^						
				Constant	60.276	2.043	29.508 (254)	<.001^c^	56.253 to 64.299
				Number of passing (CLO1)	0.735	0.303	2.429 (254)	.016^d^	0.139 to 1.332
				Instances of first-pass attempt (CLO3)	−2.570	0.572	−4.493 (254)	<.001^c^	−3.697 to −1.444
				Number of passing (CLO4)	3.520	1.219	2.888 (254)	.004^a^	1.120 to 5.921
4	0.405	0.164	<.001^c^						
				Constant	61.009	2.012	30.317 (253)	<.001^c^	57.046 to 64.972
				Number of passing (CLO1)	0.679	0.297	2.288 (253)	.023^d^	0.095 to 1.264
				Instances of first-pass attempt (CLO3)	−2.201	0.571	−3.857 (253)	<.001^c^	−3.325 to −1.077
				Number of passing (CLO4)	5.381	1.312	4.102 (253)	<.001^c^	2.797 to 7.964
				Number of total attempts (CLO4)	−1.469	0.429	−3.424 (253)	.001^a^	−2.313 to −0.624
Summative scores of course 4									
1	0.357	0.127	<.001^c^						
				Constant	32.361	6.595	4.907 (259)	<.001^c^	19.374 to 45.347
				CLO1 and 2 scores	0.454	0.074	6.150 (259)	<.001^c^	0.308 to 0.599
2	0.395	0.156	<.001^c^						
				Constant	13.440	9.095	1.478 (258)	.141	−4.470 to 31.349
				CLO1 and 2 scores	0.354	0.080	4.433 (258)	<.001^c^	0.197 to 0.512
				CLO3 scores	0.305	0.103	2.973 (258)	.003^a^	0.103 to 0.507
Rural group									
Summative scores of course 1									
1	0.482	0.232	.008^a^						
				Constant	−20.619	27.819	−0.741 (27)	.465	−77.700 to 36.462
				CLO2 scores	0.845	0.296	2.857 (27)	.008^a^	0.238 to 1.453
Summative scores of course 2									
1	0.655	0.429	<.001^c^						
				Constant	75.026	6.184	12.132 (27)	<.001^c^	62.337 to 87.715
				Instances of first-pass attempt (CLO1)	−10.182	2.261	−4.504 (27)	<.001^c^	−14.820 to −5.543
Summative scores of course 3									
1	0.441	0.194	.017^d^						
				Constant	59.697	5.668	10.532 (27)	<.001^c^	48.067 to 71.328
				Instances of highest scoring attempt (CLO4)	−5.853	2.294	-2.551 (27)	.017^d^	−10.560 to −1.145
2	0.600	0.360	.003^a^						
				Constant	57.041	5.250	10.865 (26)	<.001^c^	46.249 to 67.832
				Instances of highest scoring attempt (CLO4)	−6.060	2.086	−2.905 (26)	.007^a^	−10.347 to −1.773
				Number of passing (CLO2)	1.325	0.511	2.592 (26)	.015^d^	0.274 to 2.375
3	0.748	0.559	<.001^c^						
				Constant	54.567	4.502	12.119 (26)	<.001^c^	45.294 to 63.840
				Instances of highest scoring attempt (CLO4)	−6.331	1.767	−3.584 (25)	.001^a^	−9.969 to −2.692
				Number of passing (CLO2)	1.444	0.434	3.327 (25)	.003^a^	0.550 to 2.337
				Number of unintentional attempts (CLO1)	1.718	0.511	3.364 (25)	.002^a^	0.666 to 2.769
Summative scores of course 4									
1	0.725	0.526	<.001^c^						
				Constant	3.731	11.465	0.325 (27)	.747	−19.793 to 27.256
				CLO1 and 2 scores	0.745	0.136	5.476 (27)	<.001^c^	0.466 to 1.025

a *P*<.01.

b CLO: course learning outcome.

c *P*<.001.

d *P*<.05.

In the academic group, with summative scores as the dependent variable, positive influences included CLO scores and number of passings for each CLO, while a negative influence comprised instances of first-pass attempts (Table 5). In the quota group, a positive contribution was the number of intentional attempts, while a negative contribution was the number of total attempts (Table 5). In the test group, positive factors comprised number of passings for each CLO and CLO scores, while negative factors included instances of first-pass attempts, instances of highest scoring attempts of CLO, and number of total attempts (Table 5). In the rural group, by setting summative scores as the dependent variable, positive influences included CLO scores, number of passings for each CLO, and number of unintentional attempts, while negative contributions comprised instances of first-pass attempts and instances of highest scoring attempts of CLO (Table 5).

**Figure 3.**
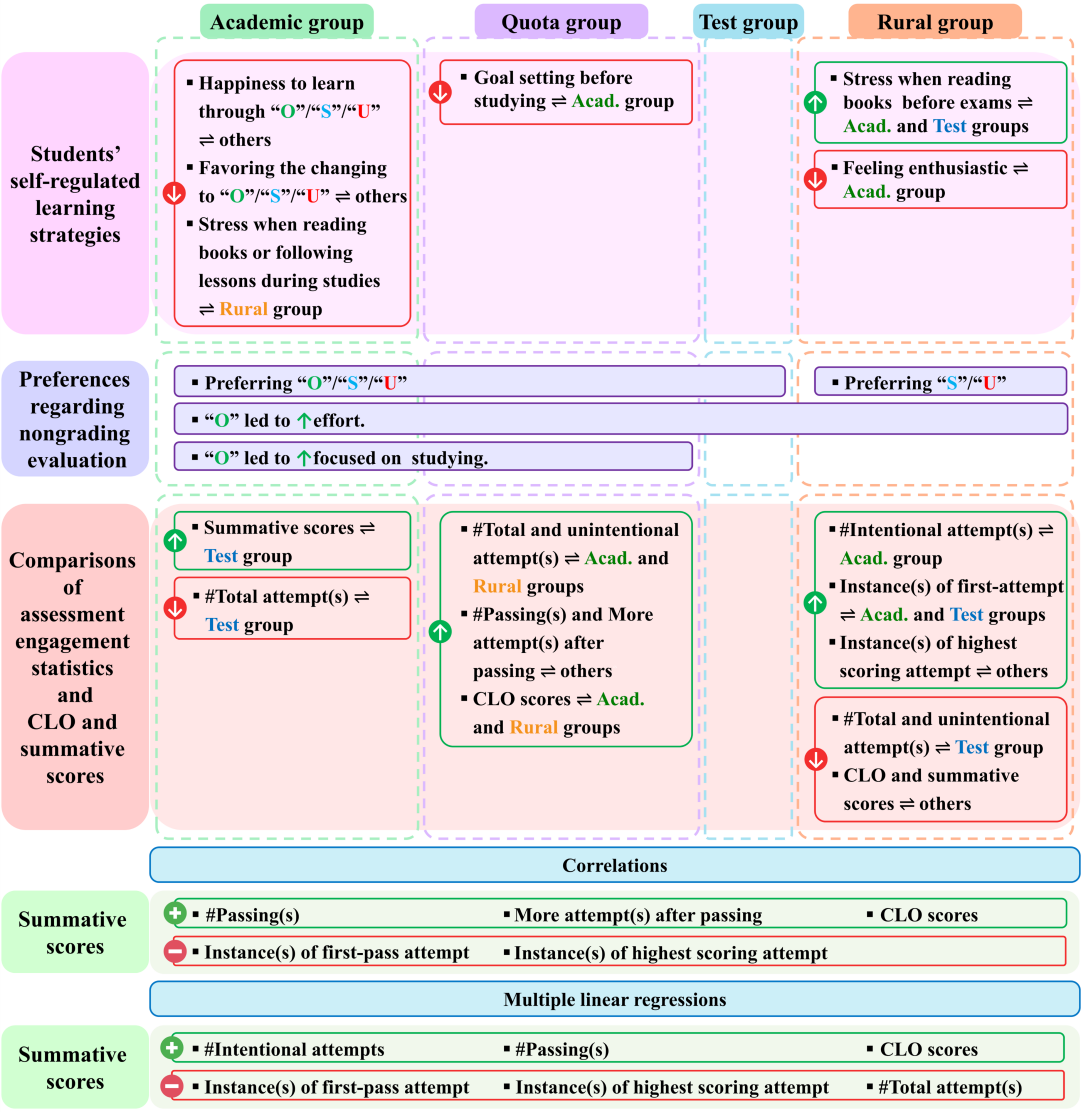
The summary of results. Acad: academic group, CLO: course learning outcome; “O”: Outstanding, others: other groups, “S”: Satisfactory, “U”: Unsatisfactory, ⇌: compared with; 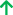: increased; 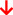: decreased; 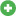: positive correlations; 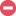: negative correlations.

Across all predictors and constant terms in the multiple linear regression models predicting summative scores, the test group generally showed narrow 95% CIs for predictors and moderately wide 95% CIs for constants, indicating overall moderate to high precision; the quota group exhibited moderate 95% CIs, reflecting moderate precision; and the academic and rural groups showed moderate to wide 95% CIs, especially wide for constants, indicating moderate to low precision (Table 5). A summary of results is shown in Figure 3.

## Discussion

### Principal Findings

This study aimed to compare SRL strategies, assessment engagement behaviors, preferences for nongrading evaluation, and academic performance across 4 admission pathways (academic, quota, test, and rural); examine correlations among summative scores, CLO scores, and engagement metrics; and identify predictors of summative scores using multiple linear regression. In line with these objectives, the main findings indicate that students in the academic group demonstrated stronger SRL strategies, fewer but more effective assessment engagement behaviors, and higher CLO and summative scores; and the rural group exhibited lower SRL, greater academic obstacles, higher numbers of assessment attempts, and the lowest academic performance, while the quota and test groups showed intermediate patterns. Most academic, quota, and test students preferred “‘O”/“S”/“U,” whereas rural students favored “S”/“U.” Summative scores exhibited positive correlations with CLO scores and number of passings, whereas they showed negative correlations with first-pass attempts. In the multiple linear regression analyses, summative scores were contributed positively by number of passings and CLO scores, whereas they were predicted negatively by instances of first-pass attempts and highest scoring attempts.

When comparing SRL strategies across admission pathways, students in the academic group showed stronger goal setting and greater enthusiasm for learning activities than other groups. They also reported less stress during routine study, whereas rural students experienced more stress before examinations than their peers.

Basically, the academic group, composed of science Olympiads students who participate in science competitions, generally demonstrates a high interest in science and learning, comes from competitive backgrounds, and possesses higher levels of general cognitive abilities [29]. Furthermore, they typically have a self-concept of ability [30], self-efficacy [29], short-term and long-term goals [29], perseverance [31], and self-discipline [31], all indicative of SRL [29,32]. In addition, they usually have support from peers, family, and teachers [29,33]. Therefore, it is not surprising that they exhibited higher levels of active goal setting and enthusiasm, showed less stress, and were more likely to receive higher scores. This finding aligns with recent research showing that medical graduates from more educationally privileged backgrounds (ie, those who attended fee-paying schools) were significantly more likely to succeed in postgraduate medical examinations [34]. In contrast, the rural group, typically from less competitive backgrounds, rated lower levels of enthusiasm but higher levels of stress when learning alongside students from high academic backgrounds [35]. These students may face unique challenges, such as limited access to educational resources and support systems [35-37].

Interestingly, students in the academic group were less satisfied with learning through nongrading evaluation and less supportive of adopting it for all preclinical courses than other groups. Most students in the academic, quota, and test pathways favored the inclusion of the “O” grade because they felt that it encouraged focus and effort, whereas rural students showed a slight preference for the simpler “S”/“U” system. These results reflect that the academic group might have familiarity with competitive academic environments [29,32] and a preference for evaluation systems that differentiate performance and offer visible recognition of achievement. The “O” grade appears to serve as an external motivating factor by reinforcing effort and focus through a tangible reward for students in most groups [10]. Students in the rural group showed a slight preference for the simpler “S”/“U” system, which was possibly due to a desire to reduce pressure or stress associated with competitive assessments, or differing motivational orientations shaped by prior educational experiences [35-37].

For assessment engagement and academic performance, students in the academic group achieved higher CLO and summative scores and completed fewer attempts overall, including early passes and highest scoring attempts, compared with other groups. These results suggest that the academic group, with a science Olympiads background, had strong SRL abilities that contributed to efficient learning and better outcomes.

In contrast, rural students showed lower CLO and summative scores and needed more attempts to pass each CLO, indicating greater challenges in regulating their learning, which may stem from factors that hinder their ability to effectively manage learning [36]. These results are consistent with a previous study in Jordan, which demonstrated that students from remote areas performed worse than those admitted through the competitive pathway or the academically excellent track [1].

In the correlation analysis of summative scores, the number of passings for each CLO, additional attempts after passing, and CLO scores consistently showed positive associations, whereas instances of first-pass attempts and highest scoring attempts showed negative associations. Similarly, in the multiple linear regression analyses, the number of intentional attempts, number of passings, and CLO scores contributed positively to summative scores, whereas total attempts, first-pass attempts, and highest scoring attempts contributed negatively. Taken together, these findings indicate that formative mastery—rather than the frequency of attempts—is the strongest predictor of summative success. This interpretation aligns with prior research showing that repeated unsuccessful attempts often reflect ineffective regulation or shallow processing strategies, which in turn predict lower achievement [38,39].

These empirical patterns align well with theoretical perspectives from Zimmerman’s cyclical SRL model, in which the Forethought Phase—comprising goal setting and strategic planning—shapes subsequent performance behaviors [7]. Students requiring multiple first-pass attempts or repeated highest scoring attempts may reflect weaker planning or monitoring processes—consistent with evidence that inadequate forethought predicts inefficient learning behaviors and lower achievement [7,39]. Conversely, the positive contributions of CLO scores and purposeful engagement align with findings that according to Zimmerman’s SRL model, self-monitoring enables learners to detect progress and develop the efficacy needed to perform at a high level of skill, while adaptive reactions increase the effectiveness of students’ learning strategies, and self-reflection feeds forward into subsequent forethought processes [7]. Similar relationships between SRL components and academic performance have been reported in medical education, where proactive goal setting and metacognitive regulation strongly predict examination outcomes [40,41].

These interpretations are based on associations and should not be construed as evidence that modifying a single engagement metric would necessarily lead to improved summative scores without concurrent changes in broader learning strategies and support. Nevertheless, addressing these challenges requires equitable access to resources, supportive environments, and targeted instruction [42]. Our findings emphasize how differences in learning behavior and assessment engagement statistics are strongly associated with academic performance. The academic group’s pattern of fewer but more effective attempts, coupled with higher CLO and summative scores, likely reflects their stronger SRL skills. While performance-based distinctions such as the “O” grade may enhance motivation and engagement for some students, they may also exacerbate stress or feelings of disadvantage for others. To promote equity and acceptance of nongrading systems, educational strategies should include clear communication of purpose, tailored feedback, and early orientation—particularly for students less familiar with performance-based academic cultures. In contrast, the rural group required more attempts and demonstrated lower performance, suggesting a need for earlier support and closer monitoring. These insights underscore the importance of using assessment engagement metrics as early indicators for identifying at-risk students. Interventions such as targeted mentoring, structured remediation, and proactive counseling, along with curriculum designs that promote SRL, may help foster equitable academic success across diverse student populations.

### Limitations

This study has several limitations. First, there were disparities in sample sizes across admission groups, particularly in the quota group, which included only 6 students. This small number may have limited the statistical power of comparisons and correlation analyses within and between groups. In addition, a few students in the quota group exhibited an unusually high number of CLO attempts, contributing to outliers that may have affected the robustness and generalizability of the findings, as reflected by the very wide 95% CIs observed in this group, indicating low precision due to high variability and the small sample size. Second, the number of allowed attempts for CLO assessments varied across courses; notably, only 1 attempt was permitted for course 4, which limited the ability to comprehensively analyze assessment engagement behaviors across all courses. Third, although descriptive demographic variables were added, including age, sex, school region, and hometown region, more detailed socioeconomic and family background information (eg, parental education, first-generation college or medical school status, and household income) was not comprehensively collected in the SiCMs. The absence of these variables limits interpretation of whether contextual factors may have contributed to the observed group differences, and future studies should incorporate such measures.

### Conclusions

Admission pathways play a crucial role in shaping students’ learning strategies, assessment engagement behaviors, and academic performance. These findings highlight the need for medical schools to adopt equity-oriented curricular approaches that not only diversify selection processes but also actively support students with varying learning profiles throughout their training. Integrating assessment-engagement metrics with SRL data provides a scalable, data-driven framework for early identification of at-risk students and enables timely, targeted interventions—such as personalized mentoring, structured remediation, and SRL-focused instructional support. Moreover, understanding group-specific preferences toward nongrading evaluation underscores the importance of designing assessment reforms that balance academic rigor with psychological safety, ensuring fairness and acceptance across diverse student cohorts, while also recognizing that SRL strategies and engagement behaviors interact with these preferences in shaping learning outcomes. Prior research often examined these factors separately [1-3], so integrating them conceptually strengthens the evidence for equity-focused curriculum design and early support strategies. In this context, our study differs from existing research by concurrently examining SRL strategies, assessment engagement behaviors, preferences regarding nongrading evaluation, and academic performance across 4 distinct admission pathways within a single preclinical cohort. This integrated analytic approach—linking engagement metrics with SRL data and performance outcomes—provides new insights into how student characteristics and learning behaviors interact within diverse admission systems. These findings generate novel, actionable knowledge for designing equity-driven educational policies and leveraging learning analytics to inform curriculum development in medical education.

## Supplementary material

10.2196/68636 Multimedia Appendix 1 Correlations of summative scores with students’ assessment engagement statistics and course learning outcome scores within the course. Blue shades represent significant positive correlations, red shades represent significant negative correlations, and gray shades represent correlation not computable due to zero variance.

10.2196/68636 Checklist 1 STROBE checklist.

10.2196/68636 Checklist 2 GRAMMS checklist.

## Abbreviations

AES: assessment engagement statistics
CLO: course learning outcome
GPA: grade point average
GRAMMS: Good Reporting of A Mixed Methods Study
MCAR: Missing Completely at Random
MMI: multiple mini-interview
O: outstanding
S: Satisfactory
SiCMs: Siriraj Campus Management System
SRL: self-regulated learning
STROBE: Strengthening the Reporting of Observational Studies in Epidemiology
TCAS: Thai University Central Admission System
TPAT: Thai Professional Aptitude Test
U: Unsatisfactory

## References

[1] Tamimi A, Hassuneh M, Tamimi I (2023). Admission criteria and academic performance in medical school. BMC Med Educ.

[2] Curtis E, Wikaire E, Jiang Y (2017). Examining the predictors of academic outcomes for indigenous Māori, Pacific and rural students admitted into medicine via two equity pathways: a retrospective observational study at the University of Auckland, Aotearoa New Zealand. BMJ Open.

[3] Aston-Mourney K, McLeod J, Rivera LR, McNeill BA, Baldi DL (2022). Prior degree and academic performance in medical school: evidence for prioritising health students and moving away from a bio-medical science-focused entry stream. BMC Med Educ.

[4] (2021). TCAS64 round 3 application guide. version 1. Council of University Presidents of Thailand.

[5] Chanmaneewong S, Yasri P (2023). Challenges faced by high school students in Thailand when preparing for medical school admissions: a comparative study of regular Thai programmes and international schools. Worldte.

[6] (2020). “Study anywhere to become a doctor, but not all are the same”: Siriraj revises medical education and transforms the medical curriculum to meet learners’ needs for the 2021 academic year, emphasizing the development of competent and compassionate physicians. Faculty of Medicine Siriraj Hospital, Mahidol University.

[7] Zimmerman BJ (2002). Becoming a self-regulated learner: an overview. Theory Pract.

[8] Villatoro Moral S, de-Benito Crosseti B (2022). Self-regulation of learning and the co-design of personalized learning pathways in higher education: a theoretical model approach. J Interactive Media Educ.

[9] Ali M, Asim H, Edhi AI (2015). Does academic assessment system type affect levels of academic stress in medical students? A cross-sectional study from Pakistan. Med Educ Online.

[10] White CB, Fantone JC (2010). Pass-fail grading: laying the foundation for self-regulated learning. Adv Health Sci Educ Theory Pract.

[11] Wilkinson TJ, Wells JE, Bushnell JA (2007). What is the educational impact of standards-based assessment in a medical degree?. Med Educ.

[12] Wilkinson T (2011). Pass/fail grading: not everything that counts can be counted. Med Educ.

[13] Hill MR, Goicochea S, Merlo LJ (2018). In their own words: stressors facing medical students in the millennial generation. Med Educ Online.

[14] Moir F, Yielder J, Sanson J, Chen Y (2018). Depression in medical students: current insights. Adv Med Educ Pract.

[15] Iyer AA, Hayes C, Chang BS (2025). Should medical school grading be tiered or pass/fail? A scoping review of conceptual arguments and empirical data. Acad Med.

[16] Sosibo L (2010). The views of academics on the use of student feedback for curriculum improvement. J Educ.

[17] Williams A (2024). Delivering effective student feedback in higher education: an evaluation of the challenges and best practice. Int J Res Educ Sci.

[18] Bendermacher GWG, De Grave WS, Wolfhagen IHAP, Dolmans DHJM, Oude Egbrink MGA (2020). Shaping a Culture for Continuous Quality Improvement in Undergraduate Medical Education. Acad Med.

[19] Broyles I, Savidge M, Schwalenberg-Leip E, Thompson K, Lee R, Sprafka S (2007). Stages of concern during curriculum change. J Int Assoc Med Sci Educ.

[20] Li IW, Jackson D (2024). Influence of entry pathway and equity group status on retention and the student experience in higher education. High Educ.

[21] Eva KW, Rosenfeld J, Reiter HI, Norman GR (2004). An admissions OSCE: the multiple mini-interview. Med Educ.

[22] Nithiapinyasakul A, Arora R, Chamnan P (2016). Impact of a 20-year collaborative approach to increasing the production of rural doctors in Thailand. Int J Med Educ.

[23] Martín Cabrera E, García LA, Torbay Betancor Á (2007). Factorial structure and reliability of a questionnaire on learning strategies in university students: CEA-U. An Psicol.

[24] Vallerand RJ, Pelletier LG, Blais MR, Briere NM, Senecal C, Vallieres EF (1992). The Academic Motivation Scale: a measure of intrinsic, extrinsic, and amotivation in education. Educ Psychol Meas.

[25] Schinske J, Tanner K (2014). Teaching more by grading less (or differently). CBE Life Sci Educ.

[26] Spring L, Robillard D, Gehlbach L, Simas TAM (2011). Impact of pass/fail grading on medical students’ well-being and academic outcomes. Med Educ.

[27] Vandenbroucke JP, von Elm E, Altman DG (2007). Strengthening the Reporting of Observational Studies in Epidemiology (STROBE): explanation and elaboration. Ann Intern Med.

[28] O’Cathain A, Murphy E, Nicholl J (2008). The quality of mixed methods studies in health services research. J Health Serv Res Policy.

[29] Tschisgale PL, Steegh A, Petersen S, Kubsch M, Wulff P, Neumann K (2024). Are science competitions meeting their intentions? a case study on affective and cognitive predictors of success in the Physics Olympiad. Discip Interdscip Sci Educ Res.

[30] Campbell JR (1996). Early identification of mathematics talent has long-term positive consequences for career contributions. Int J Educ Res.

[31] Verna MA, Feng AX (2002). American Chemistry Olympians achieve the highest level of equity. J Educ Res.

[32] Cho KK, Marjadi B, Langendyk V, Hu W (2017). The self-regulated learning of medical students in the clinical environment—a scoping review. BMC Med Educ.

[33] Campbell JR, Feng AX (2010). Comparing adult productivity of American Mathematics, Chemistry, and Physics Olympians With Terman’s Longitudinal Study. Roeper Rev.

[34] Ellis R, Knapton A, Cannon J, Lee AJ, Cleland J (2025). A multivariate analysis examining the relationship between sociodemographic differences and UK graduates’ performance on postgraduate medical exams. Med Teach.

[35] Wilkinson TJ, McKenzie JM, Ali AN, Rudland J, Carter FA, Bell CJ (2016). Identifying medical students at risk of underperformance from significant stressors. BMC Med Educ.

[36] Rodriguez F, Rivas MJ, Matsumura LH, Warschauer M, Sato BK (2018). How do students study in STEM courses? Findings from a light-touch intervention and its relevance for underrepresented students. PLoS One.

[37] Boateng BA, Thomas B (2011). How can we ease the social isolation of underrepresented minority students?. Acad Med.

[38] Alvi E, Iqbal Z, Masood F (2016). A qualitative account of the nature and use of self-regulated learning (SRL) strategies employed by university students. Aust J Teach Educ.

[39] Rovers SFE, Stalmeijer RE, van Merriënboer JJG, Savelberg HHCM, de Bruin ABH (2018). How and why do students use learning strategies? A mixed methods study on learning strategies and desirable difficulties with effective strategy users. Front Psychol.

[40] Bursalı Boz N, Oz H (2018). The role of goal setting in metacognitive awareness as a self-regulatory behavior in foreign language learning. Int Online J Educ Teach.

[41] Morisano D, Locke EA, Hattie JAC, Anderman EM (2013). International Guide to Student Achievement.

[42] O’Neal L, Perkins A (2021). Rural exclusion from science and academia. Trends Microbiol.

