# Supplementary material for: Influence of Admission Pathways on Learning Strategies, Assessment Engagement, and Academic Performance Among First-Year Medical Students: Mixed Methods Retrospective Observational and Cross-Sectional Survey Study
Source: JMIR Med Educ. 2026 Feb 2;12:e68636. doi: 10.2196/68636 (PMC12863655; doi:10.2196/68636)
Supplement: Multimedia Appendix 1 [file mededu-v12-e68636-s001.docx]

**Multimedia Appendix 1.** Correlations of summative scores with students’ assessment engagement statistics and course learning outcome scores within the course.

| **Sum scores** | **Factors** | **Academic group (N=23)** | | **Quota group (N=6)** | | **Test group (N=258)** | | **Rural group (N=29)** | |
| --- | --- | --- | --- | --- | --- | --- | --- | --- | --- |
|  |  | **R (95% CI)** | ***P*** | **R (95% CI)** | ***P*** | **R (95% CI)** | ***P*** | **R (95% CI)** | ***P*** |
| **C1** | No. of passing(s) (CLO1) | 0.580^aa^ (0.208–0.796) | .004 | 0.399 (-0.634–0.908) | .433 | 0.172^aa^ (0.050–0.287) | .006 | 0.279 (-0.102–0.582) | .143 |
|  | No. of passing(s) (CLO2) | 0.376 (-0.051–0.678) | .077 | -0.034 (-0.822–0.801) | .950 | 0.172^aa^ (0.050–0.288) | .006 | 0.213 (-0.171–0.535) | .268 |
|  | No. of passing(s) (CLO3) | 0.415^a^ (-0.006–0.702) | .049 | -0.093 (-0838–0.781) | .861 | 0.061 (-0.062–0.181) | .331 | 0.176 (-0.207–0.507) | .361 |
|  | No. of passing(s) (CLO4) | 0.321 (-0.113–0.643) | .136 | 0.186 (-0.745–0.862) | .724 | 0.178^aa^ (0.056–0.294) | .004 | 0.225 (-0.158–0.544) | .240 |
|  | No. of passing(s) (CLO5) | 0.256 (-0.181–0.600) | .239 | 0.253 (-0.716–0.877) | .629 | 0.204^aa^ (0.083–0.318) | .001 | 0.255 (-0.128–0.565) | .182 |
|  | More attempt(s) after passing (CLO1) | 0.555^aa^ (0.173–0.782) | .006 | 0.312 (-0.686–0.890) | .548 | 0.130^a^ (0.007–0.248) | .037 | 0.194 (-0.189–0.521) | .313 |
|  | More attempt(s) after passing (CLO2) | 0.390 (-0.035–0.687) | .066 | -0.034 (-0.822–0.801) | .950 | 0.173^aa^ (0.051–0.289) | .005 | 0.195 (-0.188–0.522) | .310 |
|  | More attempt(s) after passing (CLO3) | 0.415^a^ (-0.007–0.701) | .049 | -0.072 (-0.833–0.788) | .892 | 0.027 (-0.095–0.149) | .666 | 0.176 (-0.207–0.507) | .361 |
|  | More attempt(s) after passing (CLO4) | 0.295 (-0.140–0.627) | .172 | 0.186 (-0.745–0.862) | .724 | 0.161^a^ (0.039–0.278) | .010 | 0.225 (-0.158–0.544) | .240 |
|  | More attempt(s) after passing (CLO5) | 0.312 (-0.122–0.638) | .147 | 0.222 (-0.730–0.871) | .672 | 0.189^aa^ (0.068–0.304) | .002 | 0.302 (-0.078–0.598) | .111 |
|  | CLO1 scores | 0.366 (-0.063–0.671) | .086 | 0.435 (-0.610–0.914) | .389 | 0.164^aa^ (0.043–0.281) | .008 | 0.317 (-0.061–0.609) | .094 |
|  | CLO2 scores | 0.527^a^ (0.135–0.767) | .010 |  |  | 0.144^a^ (0.022–0.262) | .021 | 0.482^aa^ (0.132–0.717) | .008 |
|  | CLO3 scores | 0.601^aa^ (0.238–0.807) | .002 | 0.371 (-0.652–0.902) | .469 | 0.204^aa^ (0.084–0.318) | .001 | 0.231 (-0.152–0.548) | .229 |
|  | CLO4 scores | 0.345 (-0.086–0.658) | .107 | 0.652 (-0.395–0.951) | .161 | 0.129^a^ (0.006–0.248) | .039 | 0.436^a^ (0.074–0.688) | .018 |
|  | CLO5 scores | 0.630^aa^ (0.281–0.823) | .001 | 0.344 (-0.668–0.897) | .505 | 0.082 (-0.041–0.202) | .189 | 0.320 (-0.058–0.611) | .090 |
|  | No. of total attempts (CLO3) | 0.135 (-0.296–0.516) | .539 | -0.387 (-0.905–0.642) | .448 | -0.233^aaa^ (-0.345–(-0.113)) | <.001 | -0.111 (-0.457–0.268) | .567 |
|  | No. of total attempts (CLO4) | -0.450^a^ (-0.723–(-0.037)) | .031 | -0.392 (-0.906–0.639) | .443 | -0.168^aa^ (-0.285–(-0.046)) | .007 | 0.046 (-0.327–0.405) | .812 |
|  | No. of total attempts (CLO5) | 0.263 (-0.173–0.605) | .225 | -0.366 (-0.901–0.655) | .476 | -0.147^a^ (-0.265–(-0.025)) | .018 | 0.308 (-0.072–0.602) | .104 |
|  | No. of intentional attempt(s) (CLO3) | 0.027 (-0.390–0.434) | .903 | -0.112 (-0.843–0.774) | .832 | -0.134^a^ (-0.252–(-0.012)) | .031 | -0.070 (-0.425–0.305) | .718 |
|  | No. of unintentional attempt(s) (CLO3) | 0.174 (-0.260–0.544) | .426 | -0.377 (-0.903–0.649) | .462 | -0.208^aa^ (-0.321–(-0.088)) | .001 | -0.117 (-0.462–0.263) | .547 |
|  | No. of unintentional attempt(s) (CLO4) | -0.511^a^ (-0.758–(-0.114)) | .013 | -0.370 (-0.902–0.653) | .471 | -0.163^aa^ (-0.280–(-0.041)) | .009 | 0.138 (-0.243–0.478) | .476 |
|  | No. of unintentional attempt(s) (CLO5) | 0.003 (-0.410–0.415) | .988 | -0.466 (-0.920–0.587) | .352 | -0.208^aa^ (-0.322–(-0.087)) | .001 | 0.338 (-0.039–0.623) | .073 |
|  | Instance(s) of first-pass attempt (CLO3) | -0.437^a^ (-0.715–(-0.020) | .037 | -0.063 (-0.830–0.791) | .905 | -0.344^aaa^ (-0.447–(-0.231)) | <.001 | -0.205 (-0.529–0.178) | .287 |
|  | Instance(s) of first-pass attempt (CLO4) | -0.481^a^ (-0.740–(-0.075) | .020 | -0.024 (-0.819–0.804) | .964 | -0.158^a^ (-0.275–(-0.036)) | .012 | -0.180 (-0.511–0.202) | .349 |
|  | Instance(s) of highest scoring attempt (CLO3) | -0.133 (-0.515–0.298) | .544 | 0.027 (-0.803–0.820) | .959 | -0.160^a^ (-0.276–(-0.038)) | .010 | -0.084 (-0.436–0.293) | .666 |

| **Sum scores** | **Factors** | **Academic group (N=23)** | | **Quota group (N=6)** | | **Test group (N=258)** | | **Rural group (N=29)** | |
| --- | --- | --- | --- | --- | --- | --- | --- | --- | --- |
|  |  | **R (95% CI)** | ***P*** | **R (95% CI)** | ***P*** | **R (95% CI)** | ***P*** | **R (95% CI)** | ***P*** |
| **C2** | No. of total attempts (CLO1) | 0.136 (-0.295–0.517) | .536 | 0.814^a^ (-0.075–0.975) | .049 | 0.032 (-0.091–0.153) | .613 | -0.359 (-0.638–0.015) | .056 |
|  | No. of passing(s) (CLO2) | 0.279 (-0.157–0.616) | .198 | 0.719 (-0.290–0.961) | .108 | 0.232^aaa^ (0.113–0.344) | <.001 | 0.146 (-0.236–0.484) | .451 |
|  | No. of passing(s) (CLO3) | 0.289 (-0.146–0.623) | .181 | -0.240 (-0.875–0.721) | .647 | 0.233^aaa^ (0.113–0.345) | <.001 | 0.364 (-0.009–0.641) | .052 |
|  | More attempt(s) after passing (CLO1) | 0.117 (-0.313–0.503) | .595 | 0.794 (-0.128–0.972) | .059 | 0.125^a^ (0.003–0.243) | .045 | -0.158 (-0.494–0.224) | .413 |
|  | More attempt(s) after passing (CLO2) | 0.279 (-0.157–0.616) | .198 | 0.719 (-0.290–0.961) | .108 | 0.209^aa^ (0.088–0.322) | .001 | 0.136 (-0.245–0.477) | .481 |
|  | More attempt(s) after passing (CLO3) | 0.289 (-0.146–0.623) | .181 | -0.240 (-0.875–0.721) | .647 | 0.198^aa^ (0.076–0.313) | .001 | 0.356 (-0.019–0.635) | .058 |
|  | CLO1 scores | 0.340 (-0.091–0.656) | .112 | 0.511 (-0.550–0.928) | .300 | 0.144^a^ (0.022–0.261) | .021 | 0.325 (-0.053–0.614) | .085 |
|  | CLO2 scores | 0.085 (-0.341–0.479) | .700 |  |  | 0.134^a^ (0.011–0.251) | .032 | 0.188 (-0.195–0.517) | .328 |
|  | CLO3 scores | 0.449^a^ (0.035–0.722) | .032 | 0.259 (-0.712–0.879) | .620 | 0.253^aaa^ (0.134–0.364) | <.001 | 0.225 (-0.158–0.544) | .241 |
|  | CLO4 scores | 0.432^a^ (0.014–0.712) | .040 | 0.533 (-0.530–0.932) | .276 | 0.196^aa^ (0.075–0.312) | .002 | 0.296 (-0.085–0.594) | .119 |
|  | No. of intentional attempt(s) (CLO1) | 0.139 (-0.292–0.519) | .526 | 0.926^aa^ (0.384–0.990) | .008 | 0.022 (-0.101–0.144) | .726 | -0.577^aa^ (-0.775–(-0.258)) | .001 |
|  | Instance(s) of first-pass attempt (CLO1) | 0.060 (-0.362–0.460) | .785 | 0.476 (-0.579–0.922) | .340 | -0.110 (-0.229–0.013) | .079 | -0.655^aaa^ (-0.820–(-0.370)) | <.001 |
|  | Instance(s) of first-pass attempt (CLO2) | -0.332 (-0.650–0.101) | .122 | 0.233 (-0.725–0.873) | .657 | -0.123^a^ (-0.241–0.000) | .049 | -0.395^a^ (-0.661–(-0.026)) | .034 |
|  | Instance(s) of first-pass attempt (CLO3) | -0.491^a^ (-0.746–(-0.088) | .017 | -0.783 (-0.971–0.155) | .065 | -0.260^aaa^ (-0.371–(-0.141)) | <.001 | -0.441^a^ (-0.691–(-0.082)) | .017 |
|  | Instance(s) of first-pass attempt (CLO4) | -0.136 (-0.517–0.296) | .537 | -0.451 (-0.917–0.598) | .369 | -0.244^aaa^ (-0.356–(-0.124)) | <.001 | -0.359 (-0.638–0.015) | .055 |
|  | Instance(s) of highest scoring attempt (CLO1) | 0.106 (-0.323–0.494) | .631 | 0.730 (-0.270–0.963) | .100 | -0.014 (-0.136–0.108) | .818 | -0.614^aaa^ (-0.797–(-0.310)) | <.001 |
|  | Instance(s) of highest scoring attempt (CLO4) | -0.041 (-0.445–0.378) | .851 | -0.169 (-0.858–0.752) | .748 | -0.153^a^(-0.271–(-0.030)) | .014 | -0.192 (-0.520–0.191) | .317 |

| **Sum scores** | **Factors** | **Academic group (N=23)** | | **Quota group (N=6)** | | **Test group (N=258)** | | **Rural group (N=29)** | |
| --- | --- | --- | --- | --- | --- | --- | --- | --- | --- |
|  |  | **R (95% CI)** | ***P*** | **R (95% CI)** | ***P*** | **R (95% CI)** | ***P*** | **R (95% CI)** | ***P*** |
| **C3** | No. of intentional attempt(s) (CLO2) | 0.408 (-0.015–0.697) | .054 | 0.573 (-0.491–0.939) | .235 | 0.177^aa^ (0.056–0.292) | .004 | 0.078 (-0.298–0.431) | .686 |
|  | No. of unintentional attempt(s) (CLO1) | 0.114 (-0.316–0.500) | .606 | -0.078 (-0.834–0.786) | .883 | -0.024 (-0.146–0.099) | .702 | 0.393^a^ (0.024–0.660) | .035 |
|  | No. of passing(s) (CLO1) | 0.249 (-0.188–0.596) | .253 | 0.412 (-0.626–0.910) | .417 | 0.211^aa^ (0.091–0.325) | .001 | 0.300(-0.080–0.597) | .113 |
|  | No. of passing(s) (CLO2) | 0.435^a^ (0.018–0.714) | .038 | 0.797 (-0.121–0.973) | .058 | 0.214^aa^ (0.094–0.327) | .001 | 0.390^a^ (0.020–0.658) | .037 |
|  | No. of passing(s) (CLO3) | -0.351 (-0.662–0.080) | .101 | 0.277 (-0.704–0.883) | .595 | 0.170^aa^ (0.048–0.285) | .006 | 0.212 (-0.171–0.534) | .269 |
|  | No. of passing(s) (CLO4) | 0.095 (-0.332–0.486) | .668 | -0.136 (-0.850–0.765) | .798 | 0.164^aa^ (0.043–0.280) | .008 | 0.165 (-0.217–0.499) | .391 |
|  | No. of passing(s) (CLO5) | 0.251 (-0.185–0.597) | .248 | 0.381 (-0.646–0.904) | .456 | 0.200^aa^ (0.079–0.314) | .001 | 0.358 (-0.016–0.637) | .057 |
|  | More attempt(s) after passing (CLO1) | 0.233 (-0.203–0.585) | .285 | 0.173 (-0.751–0.859) | .744 | 0.150^a^ (0.028–0.267) | .016 | 0.228 (-0.155–0.546) | .233 |
|  | More attempt(s) after passing (CLO2) | 0.402 (-0.021–0.694) | .057 | 0.776 (-0.173–0.970) | .070 | 0.153^a^ (0.031–0.270) | .014 | 0.033 (-0.338–0.394) | .864 |
|  | More attempt(s) after passing (CLO3) | -0.366 (-0.672–0.063) | .086 | 0.277 (-0.704–0.883) | .595 | 0.117 (-0.006–0.235) | .061 | 0.031 (-0.340–0.392) | .874 |
|  | CLO1 scores | 0.283 (-0.153–0.619) | .191 | 0.844^a^ (0.019–0.979) | .035 | 0.173^aa^ (0.051–0.288) | .005 | 0.346 (-0.030–0.629) | .066 |
|  | CLO2 scores | 0.261 (-0.175–0.604) | .229 | -0.376 (-0.903–0.649) | .462 | 0.137^a^ (0.015–0.254) | .028 | 0.333 (-0.044–0.620) | .077 |
|  | CLO3 scores | 0.374 (-0.053–0.677) | .078 |  |  | 0.129^a^ (0.007–0.247) | .038 | 0.325 (-0.053–0.614) | .085 |
|  | CLO4 scores | 0.310 (-0.124–0.637) | .149 |  |  | 0.223^aaa^ (0.103–0.335) | <.001 | 0.511^aa^ (0.169–0.735) | .005 |
|  | CLO5 scores | 0.707^aaa^ (0.403–0.863) | <.001 |  |  | 0.137^a^ (0.015–0.255) | .027 | 0.446^a^ (0.087–0.694) | .015 |
|  | No. of total attempts (CLO3) | -0.268 (-0.609–0.168) | .217 | -0.519 (-0.929–0.543) | .292 | -0.200^aa^ (-0.314–(-0.080)) | .001 | -0.124 (-0.467–0.256) | .522 |
|  | No. of total attempts (CLO4) | -0.200 (-0.562–0.236) | .360 | -0.964^aa^ (-0.995–(-0.647) | .002 | -0.151^a^ (-0.268–(-0.030)) | .015 | -0.343 (-0.626–0.034) | .069 |
|  | No. of total attempts (CLO5) | 0.050 (-0.371–0.452) | .821 | -0.605 (-0.944–0.455) | .203 | -0.128^a^ (-0.246–(-0.005)) | .040 | 0.021 (-0.349–0.384) | .914 |
|  | No. of intentional attempt(s) (CLO4) | -0.193 (-0.557–0.242) | .378 | -0.267 (-0.881–0.709) | .609 | -0.077 (-0.197–0.046) | .218 | -0.399^a^ (-0.664–(-0.031)) | .032 |
|  | No. of unintentional attempt(s) (CLO3) | -0.150 (-0.527–0.283) | .496 | -0.595 (-0.942–0.467) | .213 | -0.202^aa^ (-0.316–(-0.081)) | .001 | 0.044 (-0.329–0.403) | .821 |
|  | No. of unintentional attempt(s) (CLO4) | -0.181 (-0.549–0.254) | .409 | -0.845^a^ (-0.980–(-0.022) | .034 | -0.148^a^ (-0.265–(-0.026)) | .018 | -0.007(-0.372–0.361) | .972 |
|  | No. of unintentional attempt(s) (CLO5) | 0.035 (-0.383–0.441) | .872 | -0.630 (-0.948–0.425) | .180 | -0.179^aa^ (-0.294–(-0.058)) | .004 | 0.055 (-0.319–0.412) | .777 |
|  | Instance(s) of first-pass attempt (CLO3) | -0.168 (-0.540–0.266) | .443 | 0.108 (-0.776–0.842) | .839 | -0.231^aaa^ (-0.343–(-0.112)) | <.001 | -0.388^a^ (-0.657–(-0.018)) | .038 |
|  | Instance(s) of first-pass attempt (CLO4) | -0.315 (-0.640–0.119) | .143 | -0.470 (-0.921–0.584) | .346 | -0.113 (-0.232–0.009) | .069 | -0.538^aa^ (-0.752–(-0.205)) | .003 |
|  | Instance(s) of highest scoring attempt (CLO4) | -0.233 (-0.585–0.203) | .285 | 0.235 (-0.724–0.874) | .654 | -0.117 (-0.235–0.006) | .061 | -0.441^a^ (-0.691–(-0.081)) | .017 |

| **Sum scores** | **Factors** | **Academic group (N=23)** | | **Quota group (N=6)** | | **Test group (N=258)** | | **Rural group (N=29)** | |
| --- | --- | --- | --- | --- | --- | --- | --- | --- | --- |
|  |  | **R (95% CI)** | ***P*** | **R (95% CI)** | ***P*** | **R (95% CI)** | ***P*** | **R (95% CI)** | ***P*** |
| **C4** | CLO1&2 scores | 0.520^a^ (0.126–0.763) | .011 | 0.239 (-0.722–0.874) | .648 | 0.357^aaa^ (0.246–0.458) | <.001 | 0.725^aaa^ (0.479–0.859) | <.001 |
|  | CLO3 scores | 0.364(-0.065–0.671) | .088 | -0.401 (-0.908–0.633) | .431 | 0.303^aaa^ (0.188–0.409) | <.001 | 0.420^a^ (0.055–0.677) | .023 |

| **R** | -0.900 | -0.800 | -0.700 | -0.600 | -0.500 | -0.400 | -0.300 | -0.200 | -0.100 | 0.000 | 0.100 | 0.200 | 0.300 | 0.400 | 0.500 | 0.600 | 0.700 | 0.800 | 0.900 |
| --- | --- | --- | --- | --- | --- | --- | --- | --- | --- | --- | --- | --- | --- | --- | --- | --- | --- | --- | --- |
|  |  |  |  |  |  |  |  |  |  |  |  |  |  |  |  |  |  |  |  |

Blue shades represent significant positive correlations. Red shades represent significant negative correlations. Grey shades represent correlation not computable due to zero variance. R=correlation coefficient, Sum=summative, C=course, No.=number, CLO=course learning outcome, ^a^*P*<.05, ^aa^*P*<.01, ^aaa^*P*<.001
