# Supplementary material for: Influence of Admission Pathways on Learning Strategies, Assessment Engagement, and Academic Performance Among First-Year Medical Students: Mixed Methods Retrospective Observational and Cross-Sectional Survey Study
Source: JMIR Med Educ. 2026 Feb 2;12:e68636. doi: 10.2196/68636 (PMC12863655; doi:10.2196/68636)
Supplement: Checklist 2 [file mededu-v12-e68636-s003.pdf]

GRAMMS checklist for mixed-methods studies

Item	Description	Page Number
Item 1	Justification for using mixed-methods design	2, 7
Item 2	Description of the mixed-methods design	2, 7-17
Item 3	How integration occurred (QUAL + QUAN)	3, 17-18, 41-46
Item 4	Limits of integration	46
Item 5	Insights from integrating methods	41-47
Item 6	Rationale for the mixing, priority, timing	2-3, 7-8, 12-16
